# Rational Design, Synthesis, and Systematic Evaluation of Redox-Responsive SN-38 Prodrugs for Selective Activation in Hypoxic Tumor Microenvironments

**DOI:** 10.3390/ph19030515

**Published:** 2026-03-21

**Authors:** Taimin Dong, Jin Xu, Xiuling Wang, Ziqiao Sun, Shuo Wang, Fanghui Chen, Hanchuang Zhu, Xinyu Zhang, Shuhai Xu, Chunguang Zheng, Dan Mao, Tianying Ren, Qiaoling Ni, Chenjing Xu, Xinyi Shen, Na Li, Dapeng Zhang, Lusha Ji, Huaizu Guo, Xuekun Wang

**Affiliations:** 1 State Key Laboratory of Macromolecular Drugs and Large-Scale Preparation, School of Pharmaceutical Sciences and Food Engineering, Liaocheng University, Liaocheng 252000, China; 2 State Key Laboratory of Macromolecular Drugs and Large-Scale Preparation, School of Pharmaceutical Sciences, Wenzhou Medical University, Wenzhou 325035, China; 3 State Key Laboratory of Macromolecular Drugs and Large-Scale Preparation, NMPA Key Laboratory for Quality Control of Therapeutic Monoclonal Antibodies, Shanghai Zhangjiang Biotechnology Co., Ltd., Shanghai 201210, China; 4 Shandong Key Laboratory of Applied Technology for Protein and Peptide Drugs, School of Pharmaceutical Sciences and Food Engineering, Liaocheng University, Liaocheng 252000, China; 5 Taizhou Mabtech Pharmaceuticals Co., Ltd., Taizhou 225316, China

**Keywords:** SN-38, topoisomerase I inhibitor, prodrug, disulfide bond, tumor microenvironment, redox-responsive

## Abstract

**Background:** The potent topoisomerase I inhibitor SN-38, the active metabolite of irinotecan, is limited in clinical application due to severe systemic toxicity. Prodrug strategies enabling selective activation in the tumor microenvironment offer a promising approach to improve its therapeutic index. This study aims to rationally design, synthesize, and systematically evaluate novel disulfide-based SN-38 prodrugs engineered for redox-responsive activation in hypoxic tumors. **Methods:** Two novel disulfide-based SN-38 prodrugs (SN-38-CSS and SN-38-LSS) were designed and synthesized; SN-38-CSS incorporates a constrained cis-piperazine-fused six-membered cyclic disulfide linker, while SN-38-LSS contains a linear disulfide tether, to differentially exploit the upregulated thioredoxin (Trx/TrxR) system in hypoxic tumor microenvironments. **Results:** Both prodrugs demonstrated high stability under physiological pH conditions and in human plasma, minimizing premature release. Crucially, they exhibited selective, rapid degradation in the presence of dithiol reductants (TCEP and DTT), mimicking Trx system activity, while remaining stable towards monothiols (GSH, L-Cys). In vitro cytotoxicity assays revealed that the prodrugs exhibited significantly reduced toxicity compared to SN-38 under normoxic conditions across most tested cell lines. However, under hypoxic conditions, their activity was significantly restored. Specifically, SN-38-CSS exhibited cytotoxicity comparable to SN-38 against MCF-7 and NCI-N87 cells, whereas SN-38-LSS showed lower activation efficiency. **Conclusions:** SN-38-CSS is identified as a promising redox and hypoxia dual-responsive prodrug candidate, highlighting the strategic use of cyclic disulfide linkers for achieving high selectivity and controlled drug release within the tumor microenvironment.

## 1. Introduction

**Figure 1:**
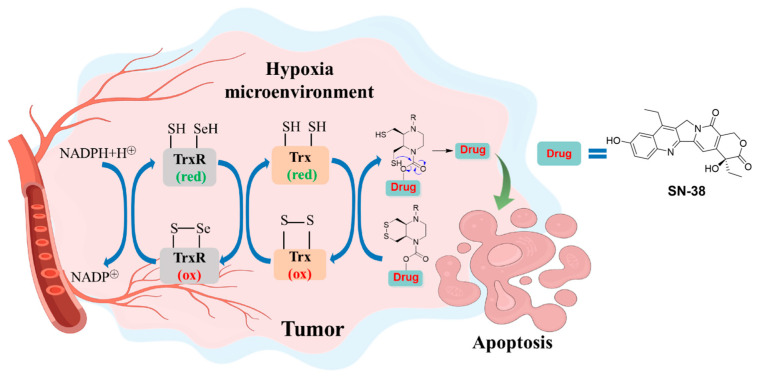
The Trx/TrxR system in the hypoxic TME and its catalytic mechanism.

Cancer remains one of the leading causes of mortality worldwide, with chemotherapy continuing to serve as a cornerstone of systemic treatment. However, the clinical utility of many potent chemotherapeutic agents is severely hampered by their lack of tumor selectivity, leading to dose-limiting systemic toxicity and adverse effects on healthy tissues [1]. The development of highly efficient, low-toxicity, and stable antineoplastic drugs has always been a topic of concern in the field of oncology [2,3,4,5,6,7]. 7-Ethyl-10-hydroxycamptothecin (SN-38) (Figure 1), the active metabolite of the prodrug irinotecan, is a potent topoisomerase I inhibitor with broad-spectrum antitumor activity [8,9]. Despite its efficacy, the clinical application of SN-38 is significantly constrained by its severe and unpredictable systemic toxicity, including life-threatening diarrhea and myelosuppression, which stem from its nonspecific distribution and activation [10,11,12]. Therefore, developing strategies to enhance the tumor-selective delivery and activation of SN-38 represents a critical unmet need in oncology.

Prodrug technology, which involves the chemical modification of an active drug into an inactive or less active form that can be selectively converted back to the parent compound at the target site, offers a powerful approach to improve the therapeutic index [13,14,15]. Ideal prodrugs should remain stable during systemic circulation to minimize off-target toxicity but undergo efficient and specific activation within the tumor microenvironment (TME) [16]. The TME possesses several distinct biochemical features that differentiate it from normal tissues, such as hypoxia, acidosis, elevated reactive oxygen species (ROS), and dysregulated redox homeostasis, providing valuable triggers for prodrug activation [17,18,19].

**Figure 2:**
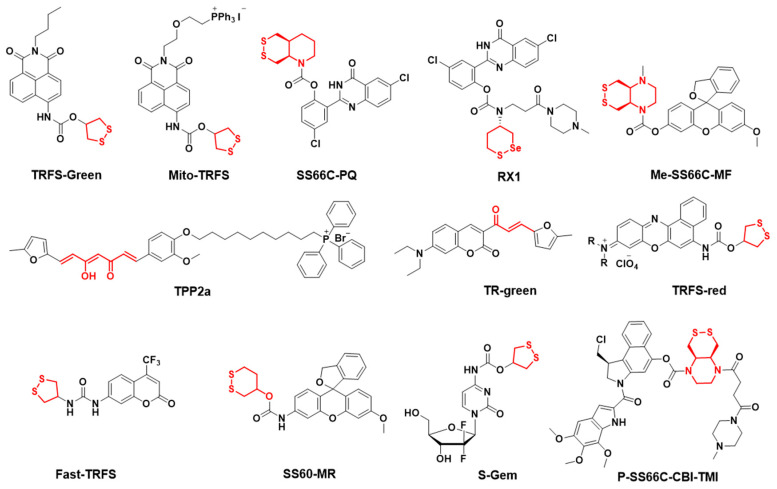
Representative fluorescent probes and prodrugs developed based on the Trx/TrxR system.

Among these features, the aberrant redox state, characterized by the overproduction of ROS and the compensatory upregulation of antioxidant systems, is particularly exploitable [19,20]. The thioredoxin (Trx) system, composed of thioredoxins (Trxs), thioredoxin reductase (TrxR), and NADPH, is a central enzymatic thiol-reducing system crucial for maintaining intracellular redox balance, DNA synthesis, and cell proliferation [21]. Notably, the Trx system is frequently overexpressed and hyperactivated in many human cancers, correlating with tumor progression, poor prognosis, and resistance to therapy [22,23,24] (Figure 1). This dysregulation offers a unique biochemical target for the design of reduction-activated prodrugs. Disulfide bonds, which are stable under oxidative or monothiol-rich conditions but susceptible to rapid cleavage by dithiol reductants like reduced Trx, have emerged as versatile linkers for constructing redox-responsive prodrugs [25,26,27,28,29]. Recent advances have demonstrated that the selectivity of disulfide cleavage can be finely tuned by their structural geometry. Specifically, constrained cyclic disulfides, such as piperazine-fused six-membered rings, exhibit remarkable stability against abundant monothiols like glutathione (GSH) while remaining highly sensitive to the dithiol activity of the Trx system, offering superior selectivity over conventional linear disulfides [30,31,32,33,34] (Figure 2).

**Figure 3:**
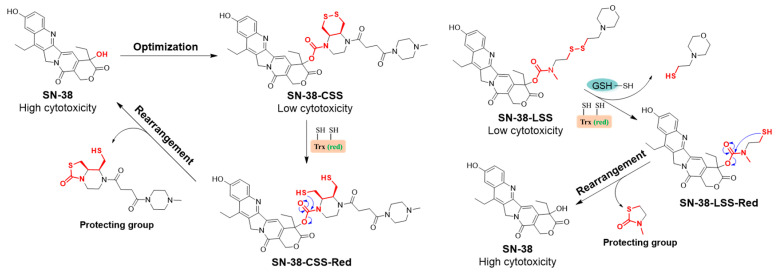
Rational design strategy and proposed metabolic activation of SN-38-CSS and SN-38-LSS.

Inspired by these principles, we herein report the rational design, synthesis, and systematic evaluation of two novel disulfide-based SN-38 prodrugs, SN-38-CSS (Figure 3) and SN-38-LSS (Figure 3), engineered for selective activation within the hypoxic TME. SN-38-CSS incorporates a sophisticated *cis*-piperazine-fused six-membered cyclic disulfide linker, designed to confer high stability in circulation and precise activation by the tumor-associated Trx system. In contrast, SN-38-LSS contains a flexible linear disulfide tether for comparative analysis. This study comprehensively investigates their chemical stability under physiological and redox-variable conditions, their plasma stability across species, and their in vitro cytotoxicity profiles under both normoxic and hypoxic conditions. Our work aims to elucidate the structure–activity relationship of disulfide linkers in prodrug design and to identify a superior candidate that effectively minimizes systemic toxicity while maximizing tumor-selective cytotoxicity through redox and hypoxia dual-responsive activation.

## 2. Results

### 2.1. Design and Chemical Synthesis of SN-38-CSS and SN-38-LSS Prodrugs

To achieve selective activation of SN-38 within the hypoxic TME via the upregulated thioredoxin (Trx/TrxR) system, two distinct disulfide-based prodrugs were rationally designed (Figure 3). SN-38-CSS incorporates a constrained *cis*-piperazine-fused six-membered cyclic disulfide linker, engineered to confer high stability against abundant monothiols (e.g., glutathione) while remaining selectively cleavable by the dithiol activity of the Trx system. In contrast, SN-38-LSS features a conventional flexible linear disulfide tether, serving as a comparative control to elucidate the impact of linker geometry on stability and activation selectivity.

**Scheme 1:**
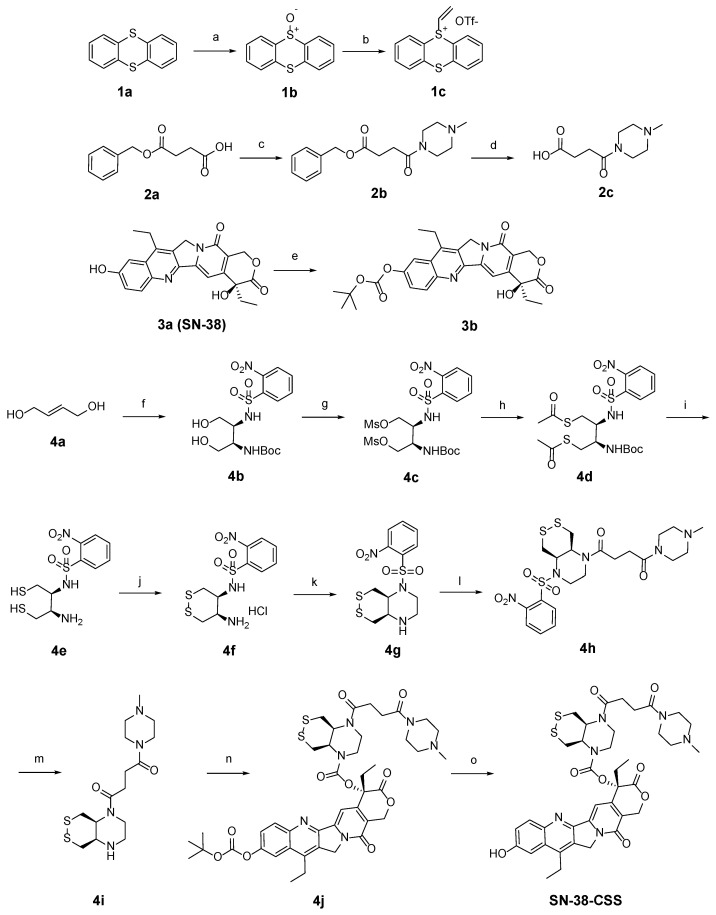
Synthesis of compound SN-38-CSS. Reagents and conditions: (a) Fe(NO_3_)_3_, NaBr, AcOH, DCM, rt, 3 h; (b) vinyltrimethylsilane, TFAA, TfOH, CH_3_CN, rt, 2 h; (c) *N*-methylpiperazine, EDCI, HOBT, DIPEA, DCM, rt, 12 h; (d) Pd/C, CH_3_OH, H_2_, rt, 2 h; (e) Boc_2_O, pyridine, DCM, rt, 12 h; (f) 2-nitrobenzenesulfonamide, BocNH_2_, I_2_, NaClO, CH_3_CN, rt, 16 h, then 45 °C, 2 h; (g) MsCl, Py, rt, 6 h; (h) KSAc, acetone, rt, 12 h; (i) HCl/1,4-dioxane, CH_3_OH, 50 °C, 14 h; (j) I_2_, rt, 4 h; (k) **1c**, DIPEA, DCM, rt, 2 h; (l) **2c**, HATU, DIPEA, DMF, rt, 2 h; (m) CH_3_ONa, MeOH, THF, rt, 1.5 h; (n) triphosgene, DMAP, DCM, 0 °C to rt, 0.5 h, then **4i**, rt, 2 h; (o) TFA/DCM, 0 °C to rt, 3 h.

The synthetic route for SN-38-CSS is depicted in Scheme 1. Thianthrene (**1a**) was oxidized by iron (III) nitrate to yield thianthrene-5-oxide (**1b**), which subsequently reacted with vinyltrimethylsilane to afford vinyl thianthrenium triflate (**1c**). 4-(Benzyloxy)-4-oxobutanoic acid (**2a**) was coupled with 1-methylpiperazine via EDCI/HOBt-mediated amide condensation to furnish the amide intermediate **2b**. Subsequent deprotection of **2b** by Pd/C-catalyzed hydrogenolysis removed the benzyl group, affording the compound 4-(4-methylpiperazin-1-yl)-4-oxobutanoic acid (**2c**) in an overall yield of approximately 66.5%. Reaction of SN-38 with Boc_2_O in the presence of pyridine afforded the corresponding 9-*O*-Boc-protected derivative **3b** in 79.7% yield [35]. A one-pot synthesis employing trans-2-butene-1,4-diol (**4a**), 2-nitrobenzenesulfonamide, and tert-butyl carbamate under iodine catalysis, with sodium hypochlorite as the oxidant, afforded the *cis*-configured intermediate **4b** in 14.3% yield. Treatment of the *cis*-configured intermediate **4b** with methanesulfonyl chloride in pyridine afforded the corresponding 1,4-bis(methanesulfonate) intermediate **4c**. Subsequent bis-nucleophilic substitution of **4c** with potassium thioacetate in acetone yielded the *cis*-configured dithioacetate intermediate **4d**. The Boc-protected intermediate **4d** was first deprotected using hydrochloric acid, followed by an iodine-catalyzed oxidative cyclization to furnish the 1,2-dithiane ring-fused sulfonamide hydrochloride **4f**. In the presence of DIPEA, intermediate **4f** underwent nucleophilic substitution/cyclization with vinyl thianthrenium salt (**1c**) to afford intermediate **4g** in 51.1% yield. Amide condensation between intermediate **4g** and intermediate **2c**, mediated by HATU and DIPEA, afforded the diamide intermediate **4h** in 57.3% yield. Subsequent removal of the *O*-nitrobenzenesulfonyl protecting group under sodium methoxide/methanol conditions yielded the key intermediate **4i** in 58.9% yield. Activation of intermediate **3b** with triphosgene, followed by condensation with intermediate **4i**, afforded the carbamate intermediate **4j.** Finally, Boc was deprotected with trifluoroacetic acid to yield the final target prodrug SN-38-CSS. The Boc deprotection afforded the target prodrug SN-38-CSS in an overall synthetic sequence that unambiguously established its structure, as confirmed by ^1^H NMR, ^13^C NMR, IR and HRMS.

**Scheme 2:**
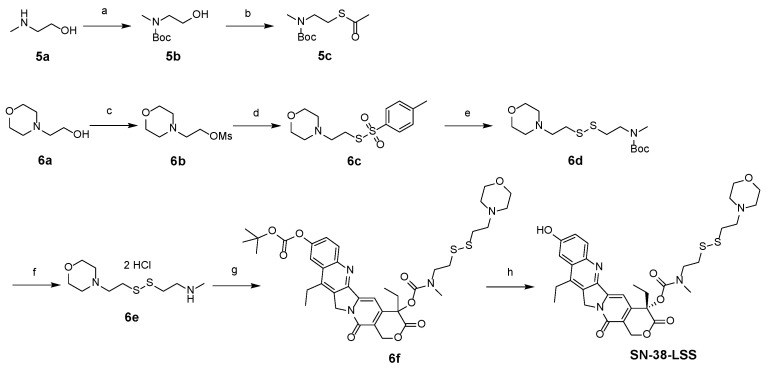
Synthesis of compound SN-38-LSS. Reagents and conditions: (a) Boc_2_O, THF, rt, 2 h; (b) PPh_3_, DIAD, HSAc, THF, 0 °C to rt, 12 h; (c) MsCl, NEt_3_, DCM, 0 °C, 30 min; (d) KSTs, acetone, 50 °C, 12 h; (e) K_2_CO_3_, MeOH, rt, 3 h; (f) TFA/DCM, rt, 2 h; (g) triphosgene, DIPEA, DCM, 0 °C to rt, 2 h; (h) TFA/DCM, rt, 2 h.

The synthetic route for the target prodrug SN-38-LSS is shown in Scheme 2. Reaction of 2-(methylamino) ethanol (**5a**) with Boc_2_O furnished compound **5b**. Subsequently, intermediate **5b** was converted to the thioacetate intermediate **5c** via a Mitsunobu reaction with thioacetic acid. Treatment of 2-morpholinoethanol (**6a**) with methanesulfonyl chloride in the presence of triethylamine yielded the corresponding methanesulfonate intermediate (**6b**). Subsequent nucleophilic substitution of **6b** with potassium *p*-toluenethiosulfonate in acetone afforded the thiosulfonate intermediate **6c**. Reaction of intermediate **6c** with intermediate **5c** in the presence of potassium carbonate, via a disulfide exchange process, afforded the disulfide-linked compound **6d** in 37.0% yield. Deprotection of the Boc group using hydrochloric acid yielded the corresponding diamine dihydrochloride **6e**. Activation of the Boc-protected SN-38 derivative **3b** with triphosgene in the presence of DMAP afforded the corresponding chloroformate intermediate. Subsequent condensation of this intermediate with **6e** yielded the carbamate intermediate **6f**. Treatment of **6f** with trifluoroacetic acid removed the Boc protecting group, furnishing the final target prodrug SN-38-LSS. Both prodrugs were obtained with high purity (≥95% by HPLC), ensuring reliable biological evaluation (Figures S29 and S38).

### 2.2. Stability Under Physiological pH Conditions

**Figure 4:**
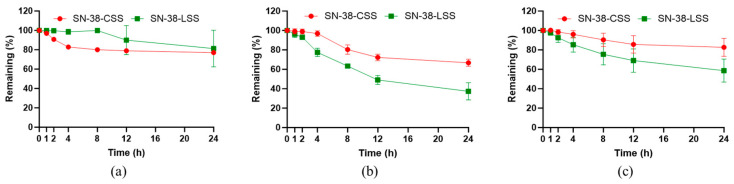
Stability of prodrugs SN-38-CSS and SN-38-LSS in PBS at different pH values. (**a**) pH 5.5, (**b**) pH 6.5, and (**c**) pH 7.4. Data represent mean ± SEM (*n* = 3).

The stability of a prodrug in systemic circulation is paramount for minimizing premature, off-target drug release [36,37,38]. We therefore assessed the stability of SN-38-CSS and SN-38-LSS in phosphate-buffered saline across a physiologically relevant pH range (5.5–7.4) over 24 h. As shown in Figure 4, both prodrugs exhibited excellent stability in phosphate buffer at pH 5.5, with >77% remaining intact and half-lives exceeding 24 h. At pH 6.5, SN-38-CSS maintained a half-life > 24 h, while SN-38-LSS showed moderate degradation (t_1/2_ ≈ 12 h). Most notably, at physiological pH 7.4, SN-38-CSS demonstrated superior stability, retaining 82.7% of its intact form after 24 h, compared to 58.7% for SN-38-LSS. These results indicate that both prodrugs, particularly SN-38-CSS, possess high resistance to nonspecific hydrolysis under conditions mimicking systemic circulation, a crucial attribute for achieving targeted delivery.

### 2.3. Plasma Stability Across Species

**Figure 5:**
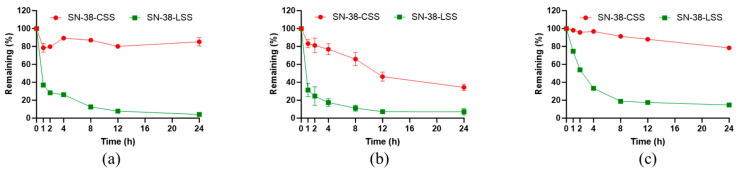
Plasma stability of prodrugs SN-38-CSS and SN-38-LSS across species, (**a**) mouse, (**b**) rat, and (**c**) human sources, incubated at 37 °C for 24 h. Data represent mean ± SEM (*n* = 3).

The stability of the prodrugs in biological matrices was evaluated in vitro using plasma from mouse, rat, and human sources (Figure 5). A pronounced interspecies variation was observed. SN-38-CSS consistently exhibited greater stability than SN-38-LSS across all species. After 24 h incubation, 85.2% (mouse), 34.3% (rat), and 78.5% (human) of SN-38-CSS remained intact. In stark contrast, SN-38-LSS degraded rapidly, with only 4.2%, 7.1%, and 14.7% remaining under the same conditions. The superior stability of SN-38-CSS, especially in human plasma, suggests a low propensity for premature activation in the bloodstream, which is expected to reduce systemic toxicity. The species-dependent degradation likely reflects differences in the concentration and activity of endogenous reducing components, highlighting an important consideration for translational research.

### 2.4. Redox-Responsive Drug Release Profiling

**Figure 6:**
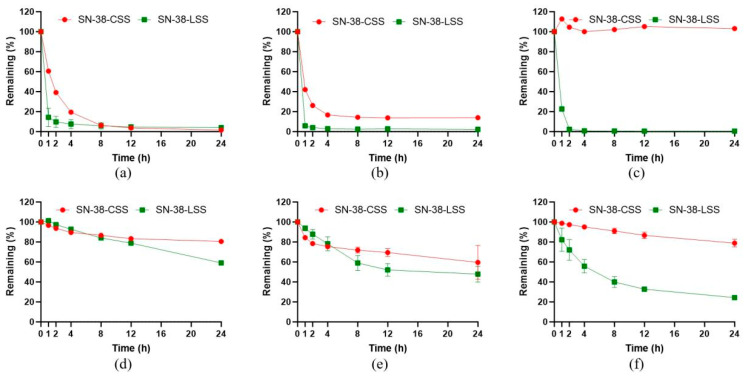
Redox-responsive degradation profiles of SN-38-CSS and SN-38-LSS in defined thiol environments: (**a**) TCEP (a dithiol reductant), (**b**) DTT (a dithiol reductant), (**c**) GSH (a monothiol), (**d**) L-Cys (a monothiol), (**e**) GSSG (oxidized glutathione), and (**f**) NADPH. Data represent mean ± SEM (*n* = 3).

The core design principle of these prodrugs is selective activation by the dithiol-based Trx system. We therefore profiled their degradation kinetics in the presence of various thiol-containing agents (Figure 6). Both prodrugs underwent rapid and nearly complete degradation within hours when exposed to the dithiol reductants TCEP or DTT, models for Trx activity, confirming efficient disulfide cleavage and SN-38 release.

Crucially, their behavior diverged significantly in the presence of monothiols. SN-38-CSS remained highly stable (>80% intact after 24 h) against physiological levels of GSH and L-cysteine (L-Cys). This demonstrates that *cis*-piperazine-fused six-membered cyclic disulfide SN-38-CSS, when reduced by monothiol compounds, rapidly undergoes intramolecular reduction to regenerate a cyclic disulfide bond [30]. It was also stable in oxidized glutathione (GSSG) and NADPH. In contrast, SN-38-LSS was rapidly degraded by GSH (t_1/2_ < 1 h) and showed considerable instability in the presence of GSH. These data underscore a critical structure–activity relationship: the constrained cyclic disulfide in SN-38-CSS confers remarkable selectivity, resisting cleavage by abundant monothiols while remaining exquisitely sensitive to dithiol reductants. This profile aligns perfectly with the goal of targeting the tumor-specific Trx system over the ubiquitous GSH background.

### 2.5. Cytotoxicity Under Normoxic Conditions (20% O_2_)

The successful masking of SN-38’s potency under normal physiological conditions is a key prodrug objective. Cytotoxicity was evaluated against a panel of six human cancer cell lines (Table 1, Figure S1). Under normoxia, both prodrugs demonstrated significantly attenuated cytotoxicity compared to parental SN-38. The IC_50_ values for SN-38-CSS were 2.6- to 15.6-fold higher than those of SN-38 across different lines, with the most pronounced reduction (15.6-fold) observed in A549 cells. SN-38-LSS showed even greater masking, with IC_50_ values 6.3- to 32.7-fold higher than SN-38. These results confirm that the disulfide-based modifications effectively reduce the inherent cytotoxicity of SN-38, indicating a strong potential to mitigate off-target effects on normal tissues during systemic circulation.

### 2.6. Cytotoxicity Under Hypoxic Conditions (5% O_2_)

Hypoxia is a hallmark of solid tumors and is known to upregulate the Trx system. We therefore assessed whether the hypoxic microenvironment could trigger the selective activation of the prodrugs (Table 2, Figure S2). Under 5% O_2_, the cytotoxicity gap between the prodrugs and SN-38 markedly narrowed. Strikingly, SN-38-CSS regained potent activity, exhibiting cytotoxicity comparable to native SN-38 in MCF-7 and NCI-N87 cells (IC_50_ ratios of 1.8 and 2.5, respectively). The results showed that in MCF-7 cells, the cytotoxicity (IC_50_) of compound SN-38-CSS was 2.41 ± 0.93 under normoxia and 0.63 ± 0.11 under hypoxia, indicating significantly enhanced cytotoxicity under hypoxic conditions. This indicates efficient hypoxia-triggered activation and drug release in these models. In contrast, while SN-38-LSS showed increased potency under hypoxia compared to normoxia, its activation was less efficient, with IC_50_ values remaining >3-fold higher than SN-38 across all cell lines. This phenomenon can be attributed to the inadequate stability of SN-38-LSS, which results in its inability to efficiently produce the active metabolite SN-38 under hypoxic conditions. The superior performance of SN-38-CSS under hypoxia underscores the advantage of its cyclic disulfide design, which likely facilitates more selective and efficient reduction by the upregulated Trx system in the hypoxic TME.

## 3. Discussion

The clinical utility of many potent chemotherapeutics, including the topoisomerase I inhibitor SN-38, is fundamentally limited by their inability to distinguish between cancerous and healthy tissues, leading to dose-limiting systemic toxicities. Prodrug strategies that leverage distinctive biochemical features of the TME for selective activation represent a compelling avenue to overcome this challenge [39]. This study was motivated by the well-established dysregulation of redox homeostasis in tumors, particularly the overexpression and hyperactivation of the Trx/TrxR system. We report the rational design and comprehensive evaluation of two novel disulfide-based SN-38 prodrugs, SN-38-CSS and SN-38-LSS, engineered to exploit this redox imbalance. Our systematic investigation reveals a striking structure–activity relationship, wherein the geometry of the disulfide linker critically dictates prodrug stability, activation selectivity, and, ultimately, hypoxia-triggered cytotoxicity.

A cornerstone of a successful prodrug is high stability during systemic transit to prevent premature drug release and associated off-target effects. Both SN-38-CSS and SN-38-LSS demonstrated commendable stability across a physiologically relevant pH range (5.5–7.4), with minimal hydrolysis observed over 24 h. This suggests a low risk of nonspecific activation in the bloodstream or extracellular fluid. However, a more nuanced and crucial picture emerged from species-specific plasma stability assays. SN-38-CSS exhibited significantly greater stability than SN-38-LSS, particularly in human plasma, where ~78% remained intact after 24 h compared to only ~15% for the linear analogue. This pronounced difference underscores a key finding: the constrained, cyclic disulfide architecture of SN-38-CSS confers superior resistance to the complex milieu of endogenous reductants present in plasma. The rapid degradation of SN-38-LSS, likely mediated by abundant monothiols like GSH or serum proteins, highlights a potential liability of conventional linear disulfide linkers, which may lead to suboptimal pharmacokinetics and increased systemic exposure to the active payload.

The core innovation of our design lies in achieving precise redox-selective activation. The simulated redox environment assays provided definitive evidence for this selectivity. Both prodrugs were rapidly and completely degraded by dithiol reductants (TCEP, DTT), confirming the lability of the disulfide bond and efficient SN-38 release under strongly reducing conditions that mimic the activity of the Trx system. The critical divergence occurred in the presence of monothiols. SN-38-CSS remained remarkably stable (>80% intact after 24 h) against high concentrations of GSH and L-cysteine, while SN-38-LSS underwent rapid cleavage by GSH. This divergent behavior can be directly attributed to the structural design. The *cis*-fused, six-membered cyclic disulfide in SN-38-CSS presents a sterically hindered and conformationally locked motif that is poorly accessed by monothiols but remains an excellent substrate for the dithiol-active site of Trx. In contrast, the flexible linear disulfide in SN-38-LSS is readily susceptible to thiol-disulfide exchange with ubiquitous GSH. This intrinsic selectivity of the cyclic disulfide is paramount, as it ensures activation is biased towards the tumor-associated Trx system over the vast background of extracellular and intracellular GSH, thereby widening the therapeutic window.

The functional payoff of this rational design was unequivocally demonstrated in the cytotoxicity studies. Under normoxic conditions, both prodrugs showed significantly attenuated cytotoxicity across all tested cancer cell lines, confirming successful pharmacological masking of SN-38. This “de-toxification” is essential for minimizing on-target, off-tumor toxicity during circulation [40]. The true test of the prodrug strategy, however, was under hypoxic conditions (5% O_2_), a hallmark of solid tumors known to further upregulate the Trx system [41]. Here, SN-38-CSS excelled, regaining potent cytotoxicity comparable to native SN-38 in MCF-7 and NCI-N87 cells. This hypoxia-triggered “re-toxification” strongly indicates that the hypoxic TME efficiently activates the prodrug, likely through enhanced Trx system activity. In contrast, SN-38-LSS, while showing improved activity under hypoxia compared to normoxia, remained significantly less potent than SN-38. We hypothesize that this suboptimal activation stems from a combination of factors: its inherent instability may lead to partial premature degradation or non-productive side reactions before reaching the target, and its lower selectivity for the Trx system over GSH may result in less efficient activation specifically within the tumor compartment. Based on our data, we propose that the prodrugs are both hypoxia-activated and redox-responsive, and that these two mechanisms are intrinsically linked [33,42]. Under hypoxic conditions, the tumor cell’s redox balance is disrupted, leading to upregulation and hyperactivation of the thioredoxin system. This upregulated dithiol reductase activity serves as the direct trigger for prodrug activation. Therefore, the prodrugs are redox-responsive (specifically responsive to dithiol reductants), and the hypoxic environment creates the conditions for this redox activation to occur preferentially in the TME. Collectively, these data position SN-38-CSS as a superior hypoxia- and redox dual-responsive prodrug candidate, effectively translating TME-specific biochemical cues into targeted cytotoxic activity.

This study, while comprehensive in its in vitro scope, has certain limitations that chart the course for future work. First, although we evaluated the redox-responsive degradation of the prodrugs using dithiol reductants (TCEP and DTT) as models for Trx system activity, direct validation in a purified Trx/TrxR enzyme system or in Trx-overexpressing cellular models was not performed. Such experiments would provide more definitive evidence for the precise intracellular activation mechanism and kinetics mediated by the tumor-associated thioredoxin system. Second, although we evaluated the stability of the prodrugs across a physiologically relevant pH range (5.5–7.4) to simulate systemic circulation conditions, their stability under strongly acidic environments was not assessed. The primary intended application of these compounds is as payloads for Antibody–Drug Conjugates (ADCs), which are administered intravenously rather than orally; therefore, gastric stability was not a prerequisite for the current proof-of-concept study. However, should these prodrugs be developed as standalone oral therapeutics, evaluation under acidic conditions mimicking gastric fluid will be essential. Third, although we evaluated cytotoxicity across a panel of six human cancer cell lines under both normoxic and hypoxic conditions to simulate normal tissues and the tumor microenvironment, direct toxicity assessment in normal, non-cancerous cell lines was not performed. Such data would provide a more complete evaluation of the therapeutic window and safety profile of these prodrug candidates. Finally, the promising profile of SN-38-CSS must be validated in in vivo tumor-bearing models to assess its pharmacokinetics, biodistribution, maximum tolerated dose, and antitumor efficacy. The observed species-dependent plasma stability necessitates careful selection of animal models for translational studies. Furthermore, the precise intracellular kinetics of TrxR-mediated cleavage and the detailed metabolic fate of the linker moiety warrant further investigation using advanced techniques like live-cell imaging and metabolomics.

Beyond standalone prodrug application, the cyclic disulfide linker technology embodied by SN-38-CSS holds significant translational potential for broader drug delivery platforms. Its excellent stability in circulation and selective reducibility make it an ideal, traceless linker for the development of advanced therapeutic conjugates. A particularly promising direction is its integration into ADC design [40]. Incorporating this linker could lead to the next generation of “probody-drug conjugates” or “prodrug–ADCs,” where the cytotoxic payload remains inert until released specifically within the TME of the antigen-positive tumor, thereby potentially enhancing the therapeutic index of ADCs by further reducing systemic and on-target, off-tumor toxicities [14,15].

In conclusion, through rational molecular design, we have developed SN-38-CSS, a prodrug that successfully integrates high systemic stability, exquisite redox selectivity favoring the tumor-associated Trx system, and potent hypoxia-triggered activation. This work not only identifies a promising candidate for the targeted delivery of SN-38 but also provides a compelling blueprint for the use of geometrically constrained cyclic disulfides as superior linkers in redox-responsive prodrug and conjugate design, offering a strategic path to safer and more effective cancer therapies.

## 4. Materials and Methods

### 4.1. Chemical Synthesis and Characterization

All materials used were of the commercially available analytical grade without purification unless otherwise indicated. Silica gel (200–300 mesh, Qingdao Marine Chemical Company, Qingdao, China) and C18 column (Boston ODS column, Boston Scientific Corporation, Marlborough, MA, USA) were used for purification. The structures of the prodrugs SN-38-CSS and SN-38-LSS were unambiguously assessed by ^1^H NMR, ^13^C NMR, and high-resolution mass spectrometry (HRMS). ^1^H NMR and ^13^C NMR spectra were recorded using tetramethylsilane (TMS) as the internal standard in deuterated solvents, such as deuterated dimethyl sulfoxide (DMSO-*d*_6_) (Energy Chemical, Shanghai, China), deuterated chloroform (CDCl_3_) (Energy Chemical, Shanghai, China) and deuterated methanol (CD_3_OH) (Energy Chemical, Shanghai, China), with a Bruker AVANCE NEO 500 instrument (Bruker Biospin GmbH, Ettlingen, Germany) at 400 MHz and 100 MHz, respectively. The chemical shifts were reported in ppm relative to TMS as the internal standard, and coupling constants were measured in Hz. IR spectra were obtained using a Fourier transform infrared 850 (FT-IR) spectrometer (Gangdong Technology, Tianjin, China) via the KBr pellet technique. HRMS was conducted on a UPLC G2-XS QTOF spectrometer (Waters Corporation, Milford, MA, USA) with the electrospray ionization Fourier transform ion cyclotron resonance technique. The target prodrugs were ≥95% pure by HPLC analysis (Agilent Technologies, Santa Clara, CA, USA). The ^1^H NMR of key intermediates (SN-38-CSS and SN-38-LSS), the MS spectra of key intermediates (SN-38-CSS), and the NMR, HRMS, HPLC and IR spectra of the SN-38-CSS and SN-38-LSS are provided in the Supporting Materials (Figures S3–S39).

#### 4.1.1. Synthesis of Compound Thianthrene 5-Oxide (**1b**)

A suspension of thianthrene **1a** (14.0 g, 64.8 mmol) in DCM (130 mL) was sequentially charged with NaBr (0.34 g, 3.3 mmol), Fe(NO_3_)_3_ nonahydrate (26.2 g, 64.8 mmol), and AcOH (2.9 g, 48.6 mmol) and then stirred at room temperature for 3 h. The reaction mixture was diluted with water (100 mL) and extracted with DCM (150 mL × 3). The combined organic phase was washed with brine (50 mL), dried over anhydrous Na_2_SO_4_, filtered, and concentrated under pressure to give compound **1b** (13 g, 86.4% yield) as a white solid, which was used in the next step without further purification. ^1^H NMR (400 MHz, CDCl_3_) δ 7.94 (dd, *J* = 7.8, 1.0 Hz, 2H), 7.64 (dd, *J* = 7.8, 0.6 Hz, 2H), 7.57 (td, *J* = 7.6, 1.0 Hz, 2H), 7.39–7.30 (m, 2H). ESI-MS *m*/*z*: 233.1 ([M+H]^+^).

#### 4.1.2. Synthesis of Compound 5-Vinyl-5H-thianthren-5-ium Tetrafluoroborate (**1c**)

To a solution of **1b** (10.0 g, 43.0 mmol) in MeCN (400 mL) was added vinyltrimethylsilane (8.6 g, 86.0 mmol) at 0 °C, and then stirred at 0 °C for 10 min. The reaction mixture was added carefully to TFAA (18.0 mL, 130.0 mmol), followed by TfOH (4.6 mL, 52.0 mmol), and then the dark purple mixture was stirred at room temperature for 2 h. The reaction mixture was concentrated under reduced pressure to remove most of the solvent. The residue was diluted with DCM (200 mL), washed with H_2_O (100 mL × 2), NaHCO_3_ solution (100 mL × 2), and brine (20 mL × 2), dried over Na_2_SO_4_, filtered, concentrated under reduced pressure to about 20 mL, and then added to Et_2_O (200 mL). The precipitate was purified further by C18 column to give compound **1c** (6.0 g, 42.0% yield) as a white solid. ^1^H NMR (400 MHz, CDCl_3_) δ 8.33 (d, *J* = 7.4 Hz, 2H), 7.79 (d, *J* = 7.7 Hz, 2H), 7.72 (t, *J* = 7.2 Hz, 2H), 7.64 (t, *J* = 7.1 Hz, 2H), 6.68 (dd, *J* = 16.0, 8.8 Hz, 1H), 6.22 (dd, *J* = 8.8, 2.9 Hz, 1H), 6.14 (dd, *J* = 16.0, 2.9 Hz, 1H). ESI-MS *m*/*z*: 243.0 ([M+H]^+^).

#### 4.1.3. Synthesis of Compound Benzyl 4-(4-Methylpiperazin-1-yl)-4-oxobutanoate (**2b**)

To a solution of 1-methylpiperazine (4.0 g, 40.0 mmol) and 4-(benzyloxy)-4-oxobutanoic acid **2a** (8.3 g, 40.0 mmol) in DCM (70 mL) was added EDCI (11.46 g, 60 mmol), HOBT (8.1 g, 60.0 mmol), and DIPEA (25.8 g, 200.0 mmol), and then stirred at room temperature for 12 h. The reaction mixture was diluted with H_2_O (50 mL) and extracted with DCM (50 mL × 3). The combined organic phase was washed with brine and concentrated under pressure. The residue was purified by column chromatography on silica gel to give compound **2b** (7.8 g, 67.2% yield) as a colorless oil. ^1^H NMR (400 MHz, CDCl_3_) δ 7.28 (s, 5H), 5.07 (s, 2H), 3.68–3.48 (m, 2H), 3.47–3.34 (m, 2H), 2.66 (dd, *J* = 10.1, 3.7 Hz, 2H), 2.57 (dd, *J* = 10.2, 3.8 Hz, 2H), 2.36–2.25 (m, 4H), 2.23 (s, 3H). ESI-MS *m*/*z*: 291.1 ([M+H]^+^).

#### 4.1.4. Synthesis of Compound 4-(4-Methylpiperazin-1-yl)-4-oxobutanoic Acid (**2c**)

A mixture of solution **2b** (3.0 g, 10.3 mmol) and Pd/C (0.3 g) in MeOH (100 mL) was hydrogenated under a balloon pressure of H_2_ at room temperature for 2 h. The reaction mixture was filtered, and the filtrate was concentrated under reduced pressure to give compound **2c** (2.1 g, 99% yield) as a colorless oil, which was used in the next step without further purification. ^1^H NMR (400 MHz, DMSO) δ 3.47–3.32 (m, 4H), 2.50 (t, *J* = 6.4 Hz, 4H), 2.40 (d, *J* = 6.8 Hz, 2H), 2.31–2.19 (m, 4H), 2.17 (s, 3H).

#### 4.1.5. Synthesis of Compound (S)-Tert-butyl (4,11-Diethyl-4-hydroxy-3,14-dioxo-3,4,12,14-tetrahydro-1H-pyrano[3′,4′:6,7]indolizino[1,2-b]quinolin-9-yl) Carbonate (**3b**)

To a mixture of SN-38 (300.0 mg, 0.76 mmol) and Boc_2_O (332.0 mg, 1.52 mmol) in DCM (10 mL) was added pyridine (600.0 mg, 7.6 mmol), and then stirred at room temperature for 12 h. The reaction mixture was purified by C18 column to give compound **3b** (300.0 mg, 79.7% yield) as a white solid. ^1^H NMR (400 MHz, DMSO-*d*_6_) δ 8.21 (d, *J* = 9.2 Hz, 1H), 8.11 (d, *J* = 2.4 Hz, 1H), 7.74 (dd, *J* = 9.2, 2.4 Hz, 1H), 7.33 (s, 1H), 6.54 (s, 1H), 5.44 (s, 2H), 5.35 (s, 2H), 3.21 (m, 2H), 1.98–1.77 (m, 2H), 1.54 (s, 9H), 1.29 (t, 3H), 0.88 (t, 3H). ESI-MS *m*/*z*: 493.2 ([M+H]^+^).

#### 4.1.6. Synthesis of Compound Tert-butyl ((2R,3S)-1,4-Dihydroxy-3-((2-nitrophenyl)sulfonamido)butan-2-yl)carbamate (**4b**)

To a solution of 2-nitrobenzenesulfonamide (11.5 g, 56.7 mmol) and iodine (1.5 g, 5.7 mmol) in MeCN (70 mL) at 0 °C was added trans-2-butene-1,4-diol **4a** (5.0 g, 56.8 mmol) and sodium hypochlorite pentahydrate (9.4 g, 56.8 mmol) with vigorous stirring. The reaction mixture was then allowed to warm to room temperature and stirred for 16 h. After it was cooled down to 0 °C again, the reaction mixture was added to tert-butyl carbamate (10.0 g, 85.0 mmol) and sodium hypochlorite pentahydrate (14.1 g, 85.2 mmol), and then stirred at 45 °C for 2 h. The reaction mixture was quenched with brine (200 mL) and extracted with EtOAc (200 mL × 3). The combined organic layers were dried over Na_2_SO_4_, filtered, and concentrated under reduced pressure. The residue was re-suspended in DCM (80 mL) and filtered. The filtrate was concentrated under reduced pressure to give **4b** (3.3 g, 14.3% yield) as a yellow solid, which was used in the next step without further purification. ^1^H NMR (400 MHz, CDCl_3_) δ 8.12–8.10 (m, 1H), 7.93–7.91 (m, 1H), 7.81–7.75 (m, 2H), 6.29 (d, *J* = 8.8 Hz, 1H), 5.39 (d, *J* = 8.8 Hz, 1H), 4.35 (d, *J* = 8.8 Hz, 1H), 3.79 (s, 1H), 3.73 (d, *J* = 12 Hz, 1H), 3.65–3.60 (m, 2H), 3.50–3.40 (m, 1H), 3.07 (m, 1H), 2.62 (s, 1H), 1.42 (s, 9H). ESI-MS *m*/*z*: 428.1 ([M+Na]^+^).

#### 4.1.7. Synthesis of Compound (2R,3S)-2-((Tert-butoxycarbonyl)amino)-3-((2-nitrophenyl)sulfonamido)butane-1,4-diyl Dimethanesulfonate (**4c**)

To a solution of compound **4b** (3.3 g, 8.1 mmol) in pyridine (20 mL) was added MsCl (2.3 g, 20.0 mmol), and then stirred at room temperature for 6 h. Water was added to the reaction mixture, and the precipitate was filtered and washed with Et_2_O (10 mL × 2) to yield compound **4c** (3.7 g, 79.7% yield) as a brownish solid, which was used in the next step without further purification. ^1^H NMR (400 MHz, CDCl_3_) δ 8.15–8.12 (m, 1H), 7.99–7.92 (m, 1H), 7.83–7.76 (m, 2H), 6.31–6.23 (m, 1H), 5.11 (d, *J* = 7.4 Hz, 1H), 4.56 (d, *J* = 10.3 Hz, 1H), 4.39 (dd, *J* = 10.5, 3.2 Hz, 1H), 4.24–4.16 (m, 1H), 4.11–3.96 (m, 3H), 3.14 (s, 3H), 2.96 (s, 3H), 1.42 (s, 9H). ESI-MS *m*/*z*: 584.0 ([M+Na]^+^).

#### 4.1.8. Synthesis of Compound S,S′-((2R,3S)-2-((Tert-butoxycarbonyl)amino)-3-((2-nitrophenyl)sulfonamido)butane-1,4-diyl) Diethanethioate (**4d**)

To a solution of compound **4c** (3.7 g, 6.5 mmol) in acetone (80 mL) was added KSAc (3.7 g, 32.5 mmol), and then stirred at room temperature for 12 h. The reaction mixture was concentrated under reduced pressure. The residue was added to H_2_O (200 mL) and extracted with EtOAc (200 mL × 3). The combined organic phase was washed with brine and concentrated under pressure. The residue was purified by C18 column to give compound **4d** (2.2 g, 64.9% yield) as a colorless oil. ^1^H NMR (400 MHz, CDCl_3_) δ 8.17–8.10 (m, 1H), 7.96–7.92 (m, 1H), 7.80–7.76 (m, 2H), 5.98 (d, *J* = 8.8 Hz, 1H), 4.83 (d, *J* = 8.8 Hz, 1H), 3.85 (m, 1H), 3.74 (m, 1H), 3.24 (dd, *J* = 14.0, 4.0 Hz, 1H), 3.13–2.90 (m, 3H), 2.35 (s, 3H), 2.20 (s, 3H), 1.41 (s, 9H). ESI-MS *m*/*z*: 544.0 ([M+Na]^+^).

#### 4.1.9. Synthesis of Compound N-((4S,5R)-5-Amino-1,2-dithian-4-yl)-2-nitrobenzenesulfonamide Hydrochloride (**4f**)

To a solution of compound **4d** (2.2 g, 4.2 mmol) in MeOH (400 mL) was added HCl in dioxane (4.0 M, 52.5 mL, 210 mmol), and then stirred at 50 °C for 14 h. After the reaction mixture was cooled to room temperature, iodine (100.0 mg, 0.4 mmol) was added and then stirred for 4 h. The reaction mixture was concentrated under pressure. To the residue was added Et_2_O (200 mL), and the precipitate was filtered and washed with Et_2_O (100 mL) to give compound **4f** (1.3 g, 79.6% yield) as a brown solid, which was used in the next step without further purification. ^1^H NMR (400 MHz, Methanol-*d*_4_) δ 8.18 (td, *J* = 6.0, 3.3 Hz, 1H), 7.96–7.82 (m, 3H), 3.89–3.81 (m, 1H), 3.63–3.56 (m, 1H), 3.34 (s, 1H), 2.98 (dd, *J* = 14.8, 3.9 Hz, 1H), 2.81–2.66 (m, 2H), 2.56 (dd, *J* = 14.4, 8.0 Hz, 1H). ESI-MS *m*/*z*: 336.0 ([M+H]^+^).

#### 4.1.10. Synthesis of Compound (4aR,8aS)-1-((2-Nitrophenyl)sulfonyl)octahydro-[1,2]dithiino[4,5-b]pyrazine (**4g**)

To a solution of compound **4f** (1.3 g, 3.4 mmol) in DCM (50 mL) was added DIPEA (4.3 g, 33.3 mmol) and compound **1c** (2.0 g, 5.1 mmol), and then stirred at room temperature for 2 h. The reaction mixture was concentrated under reduced pressure. The residue was purified by Prep-HPLC to give compound **4g** (0.6 g, 51.1% yield) as a white solid. ^1^H NMR (400 MHz, CDCl_3_) δ 8.12–8.06 (m, 1H), 7.80–7.701 (m, 4H), 4.11 (m, 1H), 3.74–3.64 (m, 1H), 3.55–3.45 (m, 2H), 3.32 (m, 1H), 3.20 (m, 2H), 2.96 (m, 2H), 2.60 (m, 1H). ESI-MS *m*/*z*: 362.0 ([M+H]^+^).

#### 4.1.11. Synthesis of Compound 1-(4-Methylpiperazin-1-yl)-4-((4aR,8aS)-4-((2-nitrophenyl)sulfonyl)hexahydro-[1,2]dithiino[4,5-b]pyrazin-1(2H)-yl)butane-1,4-dione (**4h**)

To a mixture of compound **4g** (0.6 g, 1.7 mmol) and compound **2c** (344.0 mg, 1.7 mmol) in DMF (5 mL) was added HATU (1.0 g, 2.6 mmol) and DIPEA (1.1 g, 8.6 mmol), and then stirred at room temperature for 2 h. The resulting mixture was diluted with H_2_O (50 mL) and extracted with DCM (50 mL × 3). The combined organic phase was washed with brine and concentrated under pressure. The residue was purified by column chromatography on silica gel to give compound **4h** (540 mg, 57.3% yield) as a white solid. ^1^H NMR (400 MHz, CDCl_3_) δ 8.10–8.07 (m, 1H), 7.80–7.73 (m, 3H), 4.54 (m, 1H), 4.26 (m, 1H), 3.94–3.81 (m, 3H), 3.80–3.64 (m, 1H), 3.63–3.61 (m, 2H), 3.53–3.52 (m, 2H), 3.47–3.43 (m, 1H), 3.21–3.16 (m, 1H), 2.90–2.80 (m, 1H), 2.70–2.65 (m, 2H), 2.61–2.58 (m, 2H), 2.44–2.38 (m, 4H), 2.33 (s, 3H). ESI-MS *m*/*z*: 544.0 ([M+H]^+^).

#### 4.1.12. Synthesis of Compound 1-((4aR,8aS)-Hexahydro-[1,2]dithiino[4,5-b]pyrazin-1(2H)-yl)-4-(4-methylpiperazin-1-yl)butane-1,4-dione (**4i**)

To a solution of compound **4h** (540.0 mg, 1.0 mmol) in THF (10 mL) was added CH_3_ONa in MeOH (25%, 2.2 g, 10.0 mmol), and then stirred at room temperature for 1.5 h. The reaction mixture was quenched with water (50 mL) and extracted with EtOAc (50 mL × 3). The combined organic phase was washed with brine (50 mL), dried over anhydrous Na_2_SO_4_, filtered, and concentrated under pressure. The residue was purified by column chromatography on silica gel to give compound **4i** (210 mg, 58.9% yield) as a white solid. ^1^H NMR (400 MHz, DMSO-*d*_6_) δ 4.41 (dt, *J* = 11.6, 3.4 Hz, 1H), 4.07–3.95 (m, 1H), 3.79–3.57 (m, 1H), 3.57–3.51 (m, 1H), 3.47–3.36 (m, 5H), 3.18–3.01 (m, 2H), 3.00–2.91 (m, 2H), 2.72–2.53 (m, 3H), 2.48–2.38 (m, 2H), 2.29 (t, *J* = 4.9 Hz, 2H), 2.24–2.19 (m, 2H), 2.17 (s, 3H). ESI-MS *m*/*z*: 359.0 ([M+H]^+^).

#### 4.1.13. Synthesis of Compound (S)-9-((Tert-butoxycarbonyl)oxy)-4,11-diethyl-3,14-dioxo-3,4,12,14-tetrahydro-1H-pyrano[3′,4′:6,7]indolizino[1,2-b]quinolin-4-yl (4aS,8aR)-4-(4-(4-Methylpiperazin-1-yl)-4-oxobutanoyl)hexahydro-[1,2]dithiino[4,5-b]pyrazine-1(2H)-carboxylate (**4j**)

To a mixture of compound **3b** (200.0 mg, 0.4 mmol) and DMAP (120.0 mg, 1.0 mmol) in DCM (10 mL) was added triphosgene (48.0 mg, 0.2 mmol) at 0 °C, and then stirred at room temperature for 0.5 h. Compound **4i** (120.0 mg, 0.3 mmol) was added to the reaction mixture and then stirred at room temperature for 2 h. The reaction mixture was purified by C18 column to give compound **4j** (200.0 mg, 68.2% yield) as a yellow solid.

#### 4.1.14. Synthesis of Compound (S)-4,11-Diethyl-9-hydroxy-3,14-dioxo-3,4,12,14-tetrahydro-1H-pyrano[3′,4′:6,7]indolizino[1,2-b]quinolin-4-yl (4aS,8aR)-4-(4-(4-Methylpiperazin-1-yl)-4-oxobutanoyl)hexahydro-[1,2]dithiino[4,5-b]pyrazine-1(2H)-carboxylate (SN-38-CSS)

To a solution of compound **4j** (200.0 mg, 0.2 mmol) in DCM (10 mL) was added TFA (2.0 mL) at 0 °C, and then stirred at room temperature for 3 h. The mixture was concentrated under reduced pressure. The residue was purified by C18 column to give the target compound SN-38-CSS (100.0 mg, 56.4% yield) as a yellow solid. m.p. 226.7~227.4 °C. ^1^H NMR (400 MHz, DMSO-*d*_6_) δ 10.32 (s, 1H), 8.06–7.95 (m, 1H), 7.46–7.34 (m, 2H), 7.02 (d, *J* = 9.4 Hz, 1H), 5.52–5.40 (m, 2H), 5.35–5.23 (m, 2H), 4.49 (s, 2H), 4.15 (s, 1H), 3.89–3.56 (m, 3H), 3.54–3.36 (m, 4H), 3.25–3.00 (m, 3H), 2.94 (s, 1H), 2.55 (s, 3H), 2.48–2.38 (m, 2H), 2.36–2.01 (m, 8H), 1.29 (t, *J* = 7.6, 3H), 0.95 (t, *J* = 7.2 Hz, 3H). ^13^C NMR (100 MHz, DMSO-*d*_6_) δ 169.86, 167.88, 156.73, 159.69, 153.32, 148.56, 146.65, 143.57, 142.85, 131.43, 128.20, 127.90, 122.41, 117.21, 104.74, 94.26, 76.30, 66.08, 54.34, 53.98, 49.39, 45.15, 44.17, 40.64, 27.39, 22.24, 13.30, 7.77. IR (KBr, cm^−1^) ν: 3430, 2972, 2933, 1755, 1701, 1658, 1619, 1408, 1230, 836, 721. HRMS calcd for C_38_H_45_N_6_O_8_S_2_ [M+H]^+^ 777.2740, found 777.2742.

#### 4.1.15. Synthesis of Compound Tert-butyl (2-Hydroxyethyl)(methyl)carbamate (**5b**)

2-methylaminoethanol **5a** (1.0 g, 13.3 mmol) was dissolved in THF (10 mL), and Boc_2_O (2.9 g, 13.3 mmol) dissolved in THF (5 mL) was added dropwise at room temperature. After stirring for 2 h, the volatiles were evaporated, and the residue was taken forward to the next step without purification.

#### 4.1.16. Synthesis of Compound S-(2-((Tert-butoxycarbonyl)(methyl)amino)ethyl) Ethanethioate (**5c**)

To a solution of PPh3 (4.2 g, 16.0 mmol) in anhydrous THF (20 mL) under a nitrogen atmosphere, DIAD (3.2 g, 16.0 mmol) was added dropwise at 0 °C. After the reaction mixture was stirred for 30 min, compound **5b** (2.3 g, 13.3 mmol) in anhydrous THF (5 mL) and successively neat HSAc (1.2 g, 16.0 mmol) were added dropwise. The reaction mixture was further stirred at 0 °C for 1 h, allowed to warm to room temperature, and then further stirred for 12 h. The volatiles were removed under reduced pressure, and the residue was suspended in EtOAc (50 mL). The organic phases were washed with water (50 mL × 2) and saturated sodium chloride (30 mL × 2). The organic phase was dried with anhydrous Na_2_SO_4_, filtered, and concentrated under reduced pressure. The residue was purified by column chromatography on silica gel to give compound **5c** (2.4 g, 75.6% yield) as a yellow oil. ^1^H NMR (400 MHz, CDCl_3_) δ 3.38 (t, *J* = 7.1 Hz, 2H), 3.03 (t, *J* = 7.2 Hz, 2H), 2.92 (s, 3H), 2.36 (s, 3H), 1.49 (s, 9H).

#### 4.1.17. Synthesis of Compound 2-Morpholinoethyl Methanesulfonate (**6b**)

To a solution of 2-morpholinoethanol **6a** (1.0 g, 7.6 mmol) in anhydrous DCM (30 mL) was added Et_3_N (2.1 mL, 15.3 mmol) under N_2_ atmosphere. The mixture was cooled to 0 °C, and MsCl (0.9 mL, 11.4 mmol) was added dropwise. After stirring for 15 min, the mixture was washed with saturated NaHCO_3_ (20 mL) and dried over Na_2_SO_4_ and filtered. The volatiles were evaporated under reduced pressure, and the residue **6b** was used in the next step without purification.

#### 4.1.18. Synthesis of Compound S-(2-Morpholinoethyl) 4-Methylbenzenesulfonothioate (**6c**)

To the residue of **6b** was added acetone (30 mL) and potassium p-toluenethiosulfonate (KSTs) (1.7 g, 7.6 mmol). The mixture was warmed to reflux for 1.0 h, cooled to room temperature, and further stirred for 15 h before being filtered. The solids were washed with additional acetone and discarded. The filtrate was concentrated under reduced pressure to remove all acetone, and the residue was taken up in EtOAc (50 mL). The mixture was filtered over silica (15 g), and the silica was washed with additional EtOAc (50 mL). The volatiles were removed under reduced conditions to obtain thiosulfonate **6c** as a yellowish oil (1.8 g, 79.0% yield over 2 steps), which was used without further purification. ^1^H NMR (400 MHz, CDCl_3_) δ 7.81 (d, *J* = 8.3 Hz, 2H), 7.34 (d, *J* = 8.2 Hz, 2H), 3.71–3.62 (m, 4H), 3.14 (t, *J* = 6.8 Hz, 2H), 2.62 (t, *J* = 6.8 Hz, 2H), 2.45 (s, 3H), 2.42–2.37 (m, 4H).

#### 4.1.19. Synthesis of Compound Tert-butyl Methyl(2-((2-morpholinoethyl)disulfanyl)ethyl)carbamate (**6d**)

A solution of thiosulfonate **6c** (1.50 g, 5.0 mmol) and thioacetate **5c** (1.2 g, 5.0 mmol) in anhydrous MeOH (50 mL) was added to K_2_CO_3_ (1.4 g, 10.0 mmol) under N_2_ atmosphere at room temperature. After stirring for 5 h, the volatiles were evaporated under reduced pressure, the residue was suspended in EtOAc (30 mL) and the mixture was filtered. The solids were washed with EtOAc (10 mL) and discarded. The filtrate was concentrated under reduced pressure, and the residue was purified by column chromatography on silica gel to give compound **6d** (0.6 g, 37.0% yield). ^1^H NMR (400 MHz, CDCl_3_) δ 3.72–3.67 (m, 4H), 3.48 (t, *J* = 7.4 Hz, 2H), 2.87 (s, 3H), 2.86–2.76 (m, 4H), 2.67 (d, *J* = 8.1 Hz, 1H), 2.66 (d, *J* = 9.0 Hz, 1H), 2.49–2.44 (m, 4H), 1.44 (s, 12H).

#### 4.1.20. Synthesis of Compound N-Methyl-2-((2-morpholinoethyl)disulfanyl)ethan-1-amine Dihydrochloride (**6e**)

To a solution of disulfide **6d** (0.5 g, 1.5 mmol) and 1,4-dioxane (10 mL) was added a 4 M solution of HCl in 1,4-dioxane (2 mL). The mixture was stirred for 2 h, diluted with Et_2_O (100 mL) and filtered. The solids were washed with additional Et_2_O, and the filtrate was discarded. The solids were suspended in isopropanol (10 mL), and the mixture was warmed to reflux. After cooling back to room temperature, the mixture was diluted with isohexane (10 mL) and filtered again. The filtrate was discarded, and the solids were dried to obtain dihydrochloride **6e** (0.4 g, 85.6% yield) as a white powder, which was used in the next step without further purification. ^1^H NMR (400 MHz, DMSO-*d*_6_) δ 11.46 (s, 1H), 9.03 (s, 2H), 3.84–3.75 (m, 2H), 3.64 (t, *J* = 12.2 Hz, 2H), 3.33–3.21 (m, 4H), 3.11–3.01 (m, 4H), 2.98–2.89 (m, 4H), 2.33 (t, *J* = 2.0 Hz, 3H).

#### 4.1.21. Synthesis of Compound 9-((Tert-butoxycarbonyl)oxy)-4,11-diethyl-3,14-dioxo-3,4,12,14-tetrahydro-1H-pyrano[3′,4′:6,7]indolizino[1,2-b]quinolin-4-yl Methyl(2-((2-morpholinoethyl)disulfanyl)ethyl)carbamate (**6f**)

To a mixture of compound **3b** (0.2 g, 0.4 mmol) and DMAP (99.2 mg, 0.8 mmol) in DCM (10 mL) was added triphosgene (0.1 g, 0.4 mmol) at 0 °C, and then stirred at room temperature for 1 h. Compound **6e** (130.0 mg, 0.4 mmol) was added to the reaction mixture and then stirred at room temperature for 2 h. The mixture was diluted with DCM, and saturated NaHCO_3_ was added. The organic layers were separated and washed with brine (50 mL). The organic layers were dried over Na_2_SO_4_, filtered and concentrated under reduced pressure to afford the crude product. The crude product was purified by column chromatography to give compound **6f** (130.0 mg, 42.4% yield) as a yellow solid.

#### 4.1.22. Synthesis of Compound (S)-4,11-Diethyl-9-hydroxy-3,14-dioxo-3,4,12,14-tetrahydro-1H-pyrano[3′,4′:6,7]indolizino[1,2-b]quinolin-4-yl Methyl(2-((2-morpholinoethyl)disulfanyl)ethyl)carbamate (SN-38-LSS)

To a solution of compound **6f** (100.0 mg, 0.1 mmol) in DCM (10 mL) was added TFA (2.0 mL) at 0 °C, and then stirred at room temperature for 3 h. The mixture was concentrated under reduced pressure. The residue was purified by C18 column to give the target compound **SN-38-LSS** (46.0 mg, 53.0% yield) as a yellow solid. m.p. 226.7~227.4 °C. ^1^H NMR (400 MHz, DMSO-*d*_6_) δ 10.33 (s, 1H), 8.01 (dd, *J* = 8.9, 4.6 Hz, 1H), 7.44–7.37 (m, 2H), 7.00 (d, *J* = 30.3 Hz, 1H), 5.44 (d, *J* = 3.2 Hz, 2H), 5.27 (d, *J* = 2.4 Hz, 2H), 3.74 (t, *J* = 7.1 Hz, 1H), 3.52 (t, *J* = 4.7 Hz, 2H), 3.42 (t, *J* = 4.7 Hz, 2H), 3.16–3.03 (m, 5H), 2.98 (dd, *J* = 8.2, 6.1 Hz, 1H), 2.86–2.77 (m, 2H), 2.65 (dt, *J* = 17.6, 7.1 Hz, 2H), 2.37 (t, *J* = 4.5 Hz, 2H), 2.34–2.27 (m, 1H), 2.20–2.07 (m, 4H), 1.28 (t, *J* = 7.6 Hz, 3H), 0.93 (q, *J* = 7.4 Hz, 3H). ^13^C NMR (100 MHz, DMSO-*d*_6_) δ 167.86, 156.82, 153.74, 153.55, 148.73, 146.66, 146.52, 143.60, 142.83, 131.54, 128.26, 128.04, 122.46, 117.70, 104.83, 94.22, 75.72, 66.11, 66.00, 57.54, 57.27, 53.11, 52.89, 49.45, 47.92, 35.56, 35.20, 30.50, 22.32, 13.42, 7.86. IR (KBr, cm^−1^) ν: 3424, 3223, 2966, 2935, 1751, 1709, 1662, 1597, 1506, 1250, 1226, 1116, 835, 760. HRMS calcd for C_32_H_39_N_4_O_7_S_2_ [M+H]^+^ 655.2260, found 655.2259.

### 4.2. Stability Studies in PBS Buffers at Different pH Values

The stability of compounds SN-38-CSS and SN-38-LSS was evaluated in phosphate-buffered saline (PBS) at pH 5.5, 6.5, and 7.4. All PBS buffers contained 137 mM NaCl and 2.7 mM KCl to maintain physiological osmolarity and were prepared with analytical-grade chemicals in ultrapure water as follows: pH 7.4 buffer contained 8.1 mM Na_2_HPO_4_ and 1.47 mM KH_2_PO_4_; pH 6.5 buffer contained 14 mM Na_2_HPO_4_ and 36 mM NaH_2_PO_4_; and pH 5.5 buffer contained 5.5 mM Na_2_HPO_4_ and 44.5 mM NaH_2_PO_4_. Each buffer was prepared by dissolving the corresponding components in approximately 800 mL of ultrapure water, adjusting the pH to the target value with dilute HCl or NaOH, bringing the volume to 1 L, filtering through a 0.22 μm membrane, and storing at 4 °C until use. For the stability assay, each compound was dissolved in the respective PBS buffer (final concentration 100 μM, 1% DMSO), incubated at 37 °C, and sampled (100 μL) at 0, 1, 2, 4, 8, 12, and 24 h for analysis by high-performance liquid chromatography (HPLC). HPLC was performed on a C18 column with a gradient of mobile phase A (ultrapure water with 0.1% formic acid) and mobile phase B (acetonitrile with 0.1% formic acid) at a flow rate of 0.8 mL/min and detection at 373 nm. The gradient program was: 0–8 min, 10% to 90% B; 8–13 min, hold at 90% B; 13–18 min, return to 10% B; followed by a 3 min re-equilibration. The injection volume was 10 μL.

### 4.3. Plasma Stability Across Species

Plasma was obtained from C57BL/6 mice, SD rats, and human donors by collecting whole blood in anticoagulant tubes, followed by centrifugation at 4000× *g* for 5 min at 4 °C. For stability assessment, each compound was dissolved in the corresponding plasma (final concentration 100 µM, 1% DMSO) and incubated at 37 °C. Aliquots (100 µL) were taken at 0, 1, 2, 4, 8, 12, and 24 h, immediately mixed with 100 µL of ice-cold acetonitrile to precipitate proteins and quench the reaction, vortexed for 5 min, and centrifuged at 10,000 rpm for 10 min at 4 °C. The supernatant (10 µL) was then subjected to HPLC analysis.

### 4.4. Redox-Responsive Degradation Kinetics in Defined Thiol Environments

The reactivity of the compounds was evaluated in the presence of reagents containing one or two thiol groups. In brief, 10 μL of a DMSO stock of each compound was combined with 80 μL of TE buffer (50 mM Tris-HCl, 1 mM EDTA, pH 7.4), followed by 10 μL of one of the following solutions prepared in TE buffer: 5 mM TCEP, 10 mM DTT, 5 mM GSH, 10 mM GSSG, 10 mM NADPH, 10 mM L-Cys, or TE buffer alone (control). The final concentrations were 0.1 mM compounds together with 0.5 mM TCEP, 1 mM DTT, 0.5 mM GSH, 1 mM GSSG, 1 mM NADPH, or 1 mM L-Cys. After incubation at 37 °C, aliquots were taken at 0, 1, 2, 4, 8, 12, and 24 h, diluted with an equal volume of acetonitrile, centrifuged, and the supernatant was analyzed by HPLC. Data are expressed as mean ± mean standard error (*n* = 3).

### 4.5. In Vitro Cytotoxicity Assay

Selected cell lines (MDA-MB-231, MDA-MB-468, MCF-7, A549, NCI-N87, SK-BR-3) were harvested during the logarithmic growth phase and seeded into 96-well plates at a density of 5 × 10^3^ cells/well. All cell lines were obtained directly from the American Type Culture Collection (ATCC, Manassas, VA, USA) and cultured according to the supplier’s recommended protocols. The cells were pre-cultured for 24 h under either normoxic (37 °C, 5% CO_2_) or hypoxic (37 °C, 5% CO_2_, 5% O_2_) conditions. The target compounds (SN-38, SN-38-CSS, SN-38-LSS) were dissolved in dimethyl sulfoxide (DMSO) to prepare stock solutions, which were then serially diluted with complete medium to the desired concentrations, ensuring a final DMSO concentration of ≤0.1% in the culture medium. After the pre-culture period, the medium was replaced with fresh medium containing the test compound at the indicated concentrations. Each concentration was tested in five replicate wells, along with a solvent control (medium with 0.1% DMSO) and a blank control (cell-free medium). Following 48 h of incubation, the medium was removed, and 100 μL of fresh medium containing 10% CCK-8 reagent was added to each well. After an additional 4 h of incubation, the optical density (OD) at 450 nm was measured using a microplate reader. Cell viability was calculated as follows: Survival rate (%) = [(Drug group OD − Blank group OD)/(Solvent control group OD − Blank group OD)] × 100.

### 4.6. Statistical Analysis

All quantitative data are presented as mean ± standard error of the mean (SEM) from at least three independent experiments. Statistical comparisons were performed using GraphPad Prism software (version 9.0).

## 5. Conclusions

This study successfully designed, synthesized and systematically evaluated two novel disulfide-based SN-38 prodrugs, SN-38-CSS and SN-38-LSS, for redox-responsive activation within the hypoxic TME. The cyclic disulfide linker in SN-38-CSS conferred exceptional stability under physiological pH conditions and in human plasma, minimizing premature drug release and potential off-target toxicity. Most importantly, SN-38-CSS demonstrated exquisite redox selectivity, undergoing rapid and complete cleavage only in the presence of dithiol reductants mimicking the upregulated Trx/TrxR system, while remaining stable against abundant monothiols such as GSH. This selectivity translated directly into functional advantage: under hypoxic conditions, SN-38-CSS effectively regained cytotoxicity comparable to the parent SN-38 in specific cancer cell lines, demonstrating successful hypoxia-triggered activation. In contrast, the linear disulfide analogue SN-38-LSS showed inferior stability and activation efficiency. These findings establish SN-38-CSS as a promising redox- and hypoxia dual-responsive prodrug candidate and highlight the strategic value of geometrically constrained cyclic disulfide linkers in the rational design of tumor-selective prodrugs, offering a compelling approach to enhance the therapeutic index of potent chemotherapeutic agents like SN-38.

## Figures and Tables

**Table 1:** In vitro cytotoxicity of SN-38, SN-38-CSS, and SN-38-LSS under normoxic conditions (20% O_2_) ^a^.

Compounds	A549	MDA-MB-468	SK-BR-3	MCF-7	MDA-MB-231	NCI-N87
SN-38	0.14 ± 0.03	0.03 ± 0.01	0.03 ± 0.02	0.23 ± 0.14	0.44 ± 0.19	0.13 ± 0.12
SN-38-CSS	2.18 ± 0.45	0.39 ± 0.05	0.34 ± 0.09	2.41 ± 0.93	2.46 ± 1.04	0.34 ± 0.13
SN-38-LSS	4.58 ± 0.92	0.52 ± 0.19	0.24 ± 0.07	1.49 ± 0.55	2.77 ± 1.12	1.01 ± 0.12
difference ^b^	15.6	13.0	11.3	10.5	5.6	2.6
difference ^c^	32.7	17.3	8.0	6.5	6.3	7.8

^a^ Cells were incubated with prodrugs for 48 h, and the cytotoxicity was determined by the CCK-8 assay. The data represent the half-maximal inhibitory concentration (IC_50_) in micromolar (μM) of each compound from triplicate measurements and are expressed as mean ± SEM. ^b^ The relative potency of SN-38 and SN-38-CSS to the selected cell lines. The IC_50_ values of SN-38-CSS divided by those of SN-38 give the data on the difference. ^c^ The relative potency of SN-38 and SN-38-LSS to the selected cell lines. The IC_50_ values of SN-38-LSS divided by those of SN-38 give the data on the difference.

**Table 2:** In vitro cytotoxicity of SN-38, SN-38-CSS, and SN-38-LSS under hypoxic conditions (5% O_2_) ^a^.

Compounds	MCF-7	NCI-N87	SK-BR-3	MDA-MB-231	A549	MDA-MB-468
SN-38	0.35 ± 0.08	0.02 ± 0.00	0.17 ± 0.02	0.74 ± 0.12	0.18 ± 0.03	0.01 ± 0.00
SN-38-CSS	0.63 ± 0.11	0.05 ± 0.01	0.6 ± 0.05	3.04 ± 0.71	0.94 ± 0.27	0.08 ± 0.01
SN-38-LSS	1.79 ± 0.35	0.10 ± 0.02	0.7 ± 0.05	2.86 ± 0.49	1.21 ± 0.21	0.13 ± 0.02
difference ^b^	1.8	2.5	3.5	4.1	5.2	8.0
difference ^c^	5.1	5.0	4.1	3.9	6.7	13.0

^a^ Cells were incubated with prodrugs for 48 h, and the cytotoxicity was determined by the CCK-8 assay. The data represent the half-maximal inhibitory concentration (IC_50_) in micromolar (μM) of each prodrug from triplicate measurements and are expressed as mean ± SEM. ^b^ The relative potency of SN-38 and SN-38-CSS to the selected cell lines. The IC_50_ values of SN-38-CSS divided by those of SN-38 give the data on the difference. ^c^ The relative potency of SN-38 and SN-38-LSS to the selected cell lines. The IC_50_ values of SN-38-LSS divided by those of SN-38 give the data on the difference.

## Data Availability

The original contributions presented in this study are included in the article/Supplementary Material. Further inquiries can be directed to the corresponding authors.

## Supplementary Materials

The following supporting information can be downloaded at: https://www.mdpi.com/article/10.3390/ph19030515/s1. Supplementary Figure S1: Cytotoxicity evaluation of prodrugs SN-38-CSS and SN-38-LSS under normoxic conditions (20% O_2_). Supplementary Figure S2: Cytotoxicity evaluation of SN-38-CSS and SN-38-LSS under hypoxic conditions (5% O_2_). Supplementary Figure S3: ^1^H NMR spectrum for **1b** (CDCl_3_, 400 MHz). Supplementary Figure S4: MS spectra of **1b**. Supplementary Figure S5: ^1^H NMR spectrum for **1c** (CDCl_3_, 400 MHz). Supplementary Figure S6: MS spectra of **1c**. Supplementary Figure S7: ^1^H NMR spectrum for **2b** (CDCl_3_, 400 MHz). Supplementary Figure S8: MS spectra of **2b**. Supplementary Figure S9: ^1^H NMR spectrum for **2c** (DMSO-*d*_6_, 400 MHz). Supplementary Figure S10: ^1^H NMR spectrum for **3b** (DMSO-*d*_6_, 400 MHz). Supplementary Figure S11: MS spectra of **3b**. Supplementary Figure S12: ^1^H NMR spectrum for **4b** (DMSO-*d*_6_, 400 MHz). Supplementary Figure S13: MS spectra of **4b**. Supplementary Figure S14: ^1^H NMR spectrum for **4c** (CDCl_3_, 400 MHz). Supplementary Figure S15: MS spectra of **4c**. Supplementary Figure S16: ^1^H NMR spectrum for **4d** (CDCl_3_, 400 MHz). Supplementary Figure S17: MS spectra of **4d**. Supplementary Figure S18: ^1^H NMR spectrum for **4f** (Methanol-*d*_4_, 400 MHz). Supplementary Figure S19: MS spectra of **4f**. Supplementary Figure S20: ^1^H NMR spectrum for **4g** (CDCl_3_, 400 MHz). Supplementary Figure S21: MS spectra of **4g**. Supplementary Figure S22: ^1^H NMR spectrum for **4h** (CDCl_3_, 400 MHz). Supplementary Figure S23: MS spectra of **4h**. Supplementary Figure S24: ^1^H NMR spectrum for **4i** (DMSO-*d*_6_, 400 MHz). Supplementary Figure S25: MS spectra of **4i**. Supplementary Figure S26: ^1^H NMR spectrum for SN-38-CSS (DMSO-*d*_6_, 400 MHz). Supplementary Figure S27: ^13^C NMR spectrum for SN-38-CSS (DMSO-*d*_6_, 100 MHz). Supplementary Figure S28: HRMS spectra of SN-38-CSS. Supplementary Figure S29: HPLC spectra of SN-38-CSS. Supplementary Figure S30: IR spectra of SN-38-CSS. Supplementary Figure S31: ^1^H NMR spectrum for **5c** (CDCl_3_, 400 MHz). Supplementary Figure S32: ^1^H NMR spectrum for **6c** (CDCl_3_, 400 MHz). Supplementary Figure S33: ^1^H NMR spectrum for **6d** (CDCl_3_, 400 MHz). Supplementary Figure S34: ^1^H NMR spectrum for **6e** (DMSO-*d*_6_, 400 MHz). Supplementary Figure S35: ^1^H NMR spectrum for SN-38-LSS (DMSO-*d*_6_, 400 MHz). Supplementary Figure S36: ^13^C NMR spectrum for SN-38-LSS (DMSO-*d*_6_, 100 MHz). Supplementary Figure S37: HRMS spectra of SN-38-LSS. Supplementary Figure S38: HPLC spectrum for SN-38-LSS. Supplementary Figure S39: IR spectra of SN-38-LSS.

## Abbreviations

ADC	antibody–drug conjugate
AcOH	acetic acid
Boc_2_O	di-tert-butyl dicarbonate
CCK-8	cell counting kit-8
CH_3_CN	acetonitrile
CH_3_OH/MeOH	methanol
CH_3_ONa	sodium methoxide
DCM	dichloromethane
DIAD	diisopropyl azodicarboxylate
DMAP	4-dimethylaminopyridine
DMF	n,n-dimethylformamide
DMSO	dimethyl sulfoxide
DIPEA	n,n-diisopropylethylamine
DTT	dithiothreitol
EDCI	1-ethyl-3-(3-dimethylaminopropyl)carbodiimide
GSH	glutathione
GSSG	glutathione disulfide
HATU	1-[bis(dimethylamino)methylene]-1h-1,2,3-triazolo[4,5-b]pyridinium 3-oxide hexafluorophosphate
HPLC	high-performance liquid chromatography
HCl	hydrochloric acid
HRMS	high-resolution mass spectrometry
HOBT	1-hydroxybenzotriazole
HSAc	thioacetic acid
IC_50_	half-maximal inhibitory concentration
IR	Infrared
K_2_CO_3_	potassium carbonate
KSAc	potassium thioacetate
KSTs	potassium p-toluenethiosulfonate
L-Cys	l-cysteine
MsCl	methanesulfonyl chloride
NADPH	nicotinamide adenine dinucleotide phosphate
NEt_3_	triethylamine
NaClO	sodium hypochlorite
Pd/C	palladium on carbon
PPh_3_	triphenylphosphine
Py	pyridine
ROS	reactive oxygen species
SEM	standard error of the mean
SN-38	7-ethyl-10-hydroxycamptothecin
TCEP	tris(2-carboxyethyl)phosphine
TFAA	trifluoroacetic anhydride
TfOH	trifluoromethanesulfonic acid
TFA	trifluoroacetic acid
THF	tetrahydrofuran
t_1/2_	half-life
Trx	thioredoxin
TrxR	thioredoxin reductase
TME	tumor microenvironment
rt	room temperature
m.p.	melting point
^1^H NMR	proton nuclear magnetic resonance spectroscopy
^13^C NMR	carbon-13 nuclear magnetic resonance spectroscopy
