# Supplementary material for: Rational Design, Synthesis, and Systematic Evaluation of Redox-Responsive SN-38 Prodrugs for Selective Activation in Hypoxic Tumor Microenvironments
Source: Pharmaceuticals (Basel). 2026 Mar 21;19(3):515. doi: 10.3390/ph19030515 (PMC13029133; doi:10.3390/ph19030515)
Supplement: Supplementary file 1 [file pharmaceuticals-19-00515-s001.zip › pharmaceuticals-4171978-supplementary.pdf]

Supplementary data

Rational Design, Synthesis, and Systematic Evaluation of Redox-Responsive SN-38 Prodrugs for Selective Activation in Hypoxic Tumor Microenvironments

Taimin Dong[1,2,4,†], Jin Xu[1,2,4,†], Xiuling Wang[2,3,4], Ziqiao Sun[2,3,4], Shuo Wang[1], Fanghui Chen[3], Hanchuang Zhu[3], Xinyu Zhang[1,2,4], Shuhai Xu[1,2,4], Chunguang Zheng[1,2,4], Dan Mao[1,2,4], Tianying Ren[1,2,4], Qiaoling Ni[1,2,4], Chenjing Xu[1,2,4], Xinyi Shen[1,2,4], Na Li[1,2,4], Dapeng Zhang[1,2,4,5], Lusha Ji[1,2,4], Huaizu Guo[1,2,4,*], Xuekun Wang[1,2,4,*]

[1] State Key Laboratory of Macromolecular Drugs and Large-Scale Preparation, School of Pharmaceutical Sciences and Food Engineering, Liaocheng University, Liaocheng 252000, China

[2] State Key Laboratory of Macromolecular Drugs and Large-Scale Preparation, School of Pharmaceutical Sciences, Wenzhou Medical University, Wenzhou 325035, China

[3] State Key Laboratory of Macromolecular Drugs and Large-Scale Preparation, NMPA Key Laboratory for Quality Control of Therapeutic Monoclonal Antibodies, Shanghai Zhangjiang Biotechnology Co., Ltd., Shanghai 201210, China

[4.]Shandong Key Laboratory of Applied Technology for Protein and Peptide Drugs, School of Pharmaceutical Sciences and Food Engineering, Liaocheng University, Liaocheng 252000, China

[5] Taizhou Mabtech Pharmaceuticals Co., Ltd., Taizhou 225316, China

[*]Correspondence: guohuaizu@163.com (H.G.); xuekunwang0610@126.com (X.W.)
† These authors contributed equally to this work.

Contents:

Supplementary Figure S1. Cytotoxicity evaluation of prodrugs SN-38-CSS and SN-38-LSS under normoxic conditions (20% O_2).

Supplementary Figure S2. Cytotoxicity evaluation of SN-38-CSS and SN-38-LSS under hypoxic conditions (5% O_2).

Supplementary Figure S3-S39. The ^{1}H NMR of key intermediates (SN-38-CSS and SN-38-LSS), MS spectra of key intermediates (SN-38-CSS), and the NMR, HRMS, HPLC and IR spectra of the SN-38-CSS and SN-38-LSS.

1. Cytotoxicity evaluation of prodrugs SN-38-CSS and SN-38-LSS under normoxic conditions (20% O_2). Cells were incubated with prodrugs for 48 h, and the cytotoxicity was determined by the CCK-8 assay. The data represent the half-maximal inhibitory concentration (IC_{50}) in micromolar (μM) of each compound from triplicate measurements and are expressed as mean ± SEM.

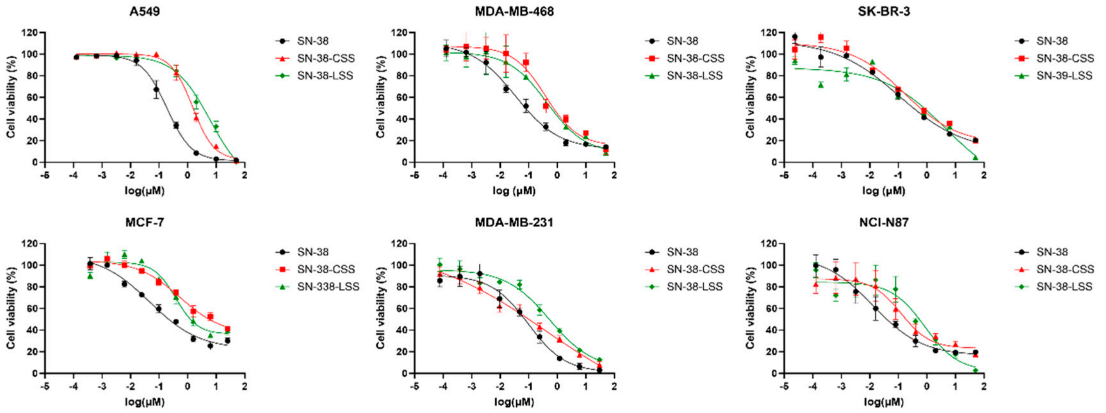

Figure S1. Cytotoxicity evaluation of prodrugs SN-38-CSS and SN-38-LSS under normoxic conditions (20% O_2)

2. Cytotoxicity evaluation of SN-38-CSS and SN-38-LSS under hypoxic conditions (5% O_2). Cells were incubated with prodrugs for 48 h, and the cytotoxicity was determined by the CCK-8 assay. The data represent the half-maximal inhibitory concentration (IC_{50}) in micromolar (μM) of each prodrugs from triplicate measurements and are expressed as mean ± SEM.

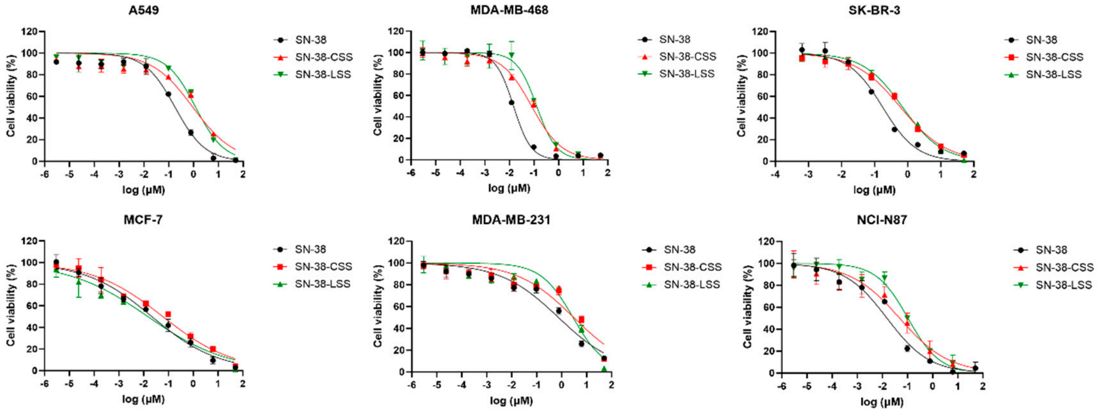

Figure S2. Cytotoxicity evaluation of SN-38-CSS and SN-38-LSS under hypoxic conditions (5% O_2)

3. Supplementary Figure S3-S39. The ^{1}H NMR of key intermediates (SN-38-CSS and SN-38-LSS), MS spectra of key intermediates (SN-38-CSS), and the NMR, HRMS, HPLC and IR spectra of the SN-38-CSS and SN-38-LSS. ^{1}H NMR and ^{13}C NMR spectra were recorded using tetramethylsilane (TMS) as the internal standard in deuterated solvents, such as deuterated dimethyl sulfoxide (DMSO-d_6), deuterated chloroform ($CDCl_3$) and deuterated methanol (CD_3OD) (Energy Chemical, Shanghai, China) with a Bruker AVANCE NEO 500 instrument (Bruker Biospin GmbH, Ettlingen, Germany) at 400 MHz and 100 MHz, respectively. The chemical shifts were reported in ppm relative to TMS as the internal standard, and coupling constants were measured in Hz. IR spectra were obtained using a Fourier transform infrared 850 (FT-IR) spectrometer (Gangdong Technology, Tianjin, China) via the KBr pellet

technique. HRMS was conducted on a UPLC G2-XS QTOF spectrometer (Waters Corporation, Milford, MA, USA) with the electrospray ionization Fourier transform ion cyclotron resonance technique. The target prodrugs were ≥95% pure by HPLC analysis (Agilent Technologies, Santa Clara, CA, USA).

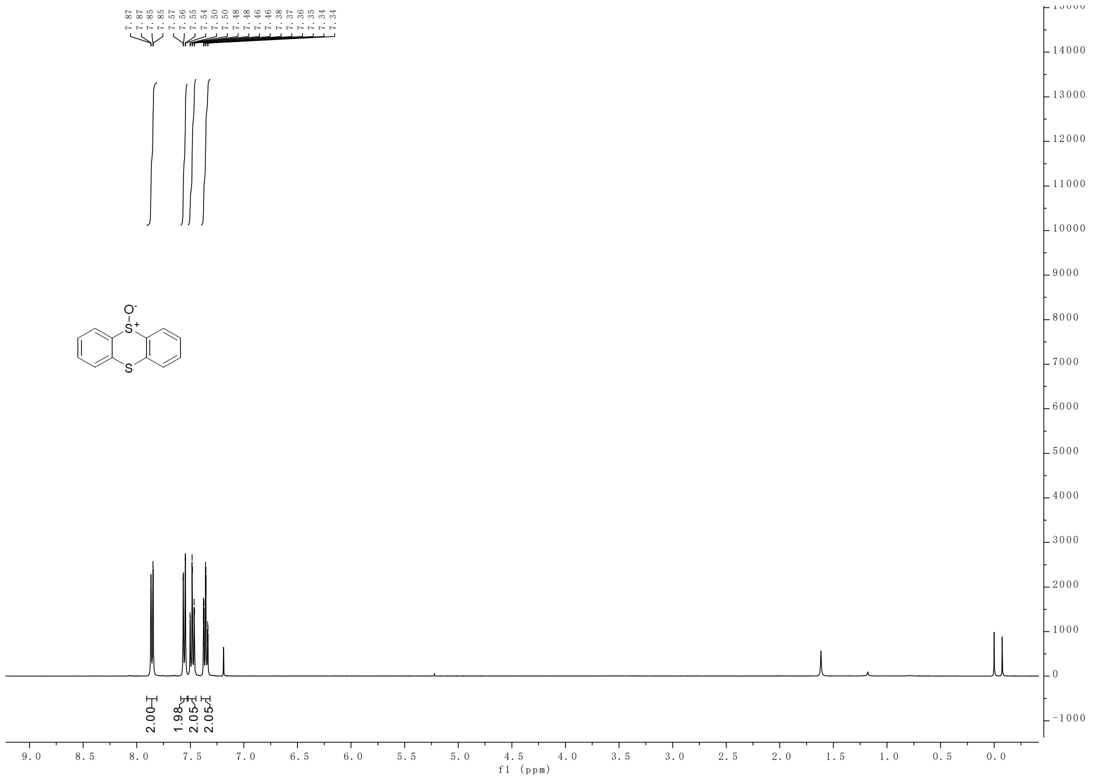

Figure S3. ^{1}H NMR spectrum for **1b** ($CDCl_3$, 400 MHz)

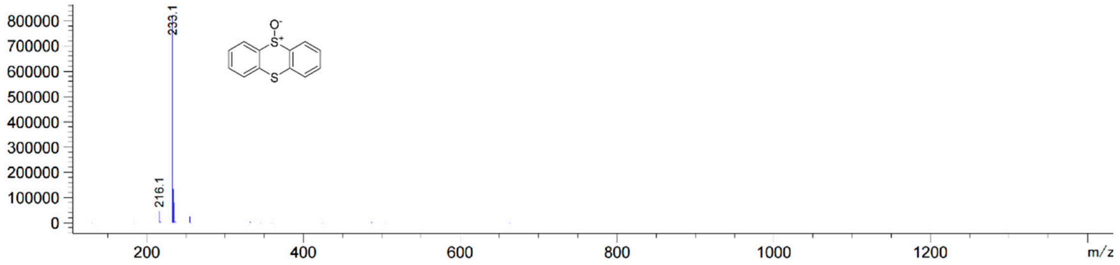

Figure S4. MS spectra of **1b**

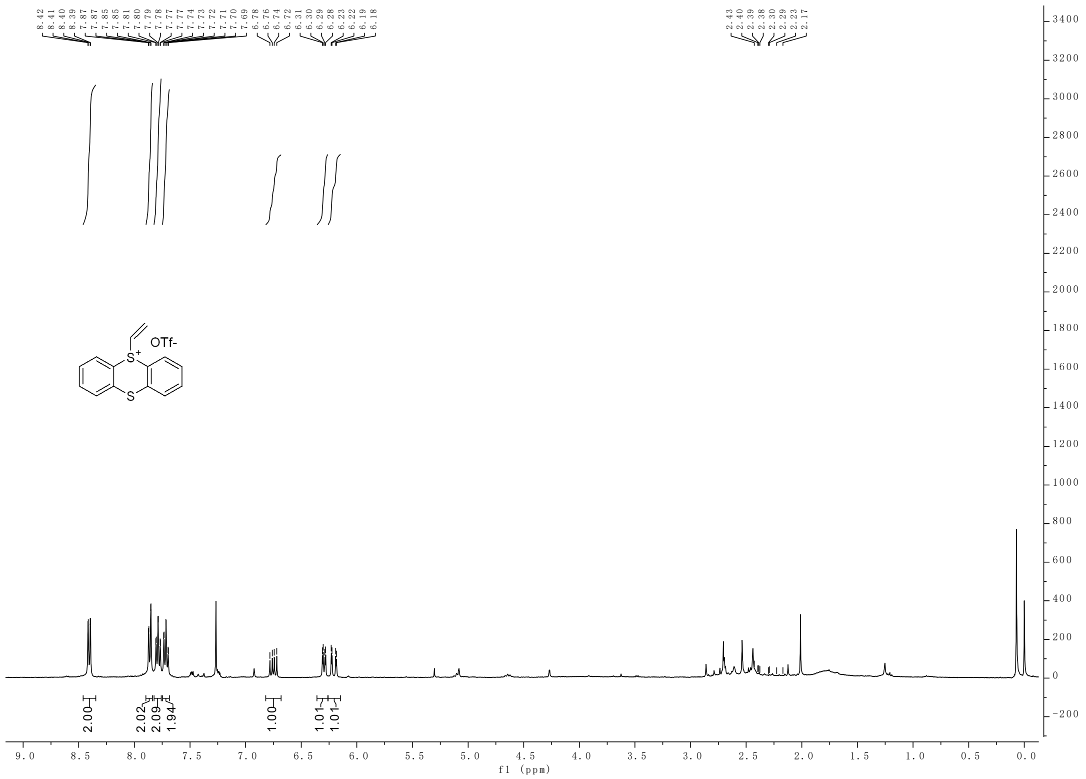

Figure S5. ^{1}H NMR spectrum for **1c** ($CDCl_3$, 400 MHz)

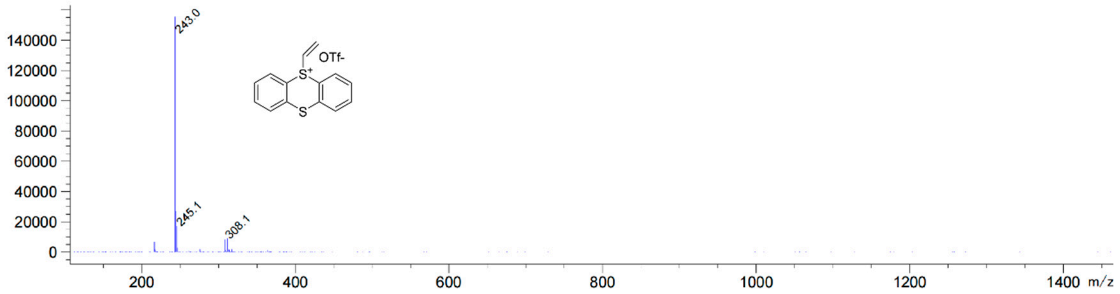

Figure S6. MS spectra of **1c**

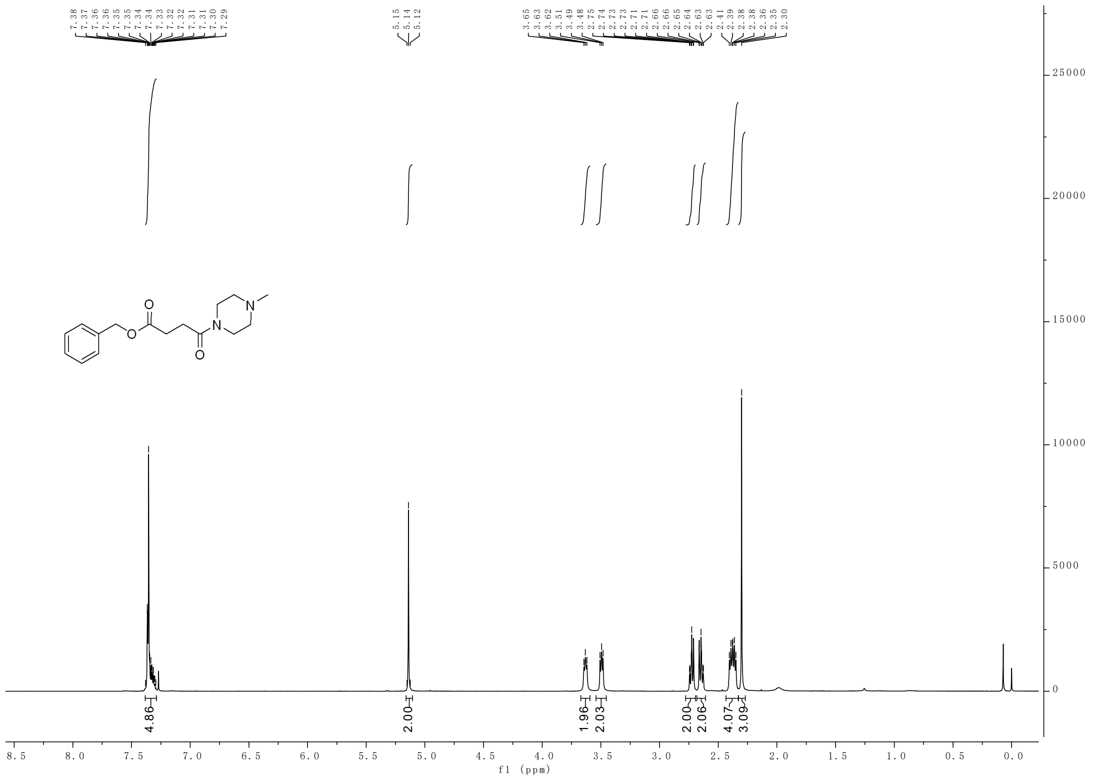

Figure S7. ^{1}H NMR spectrum for **2b** ($CDCl_3$, 400 MHz)

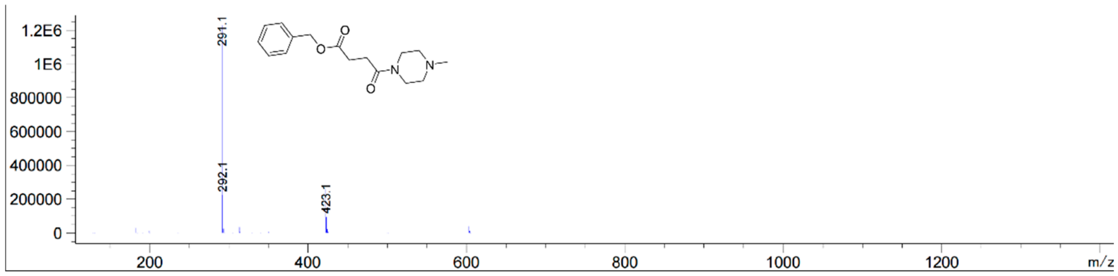

Figure S8. MS spectra of **2b**

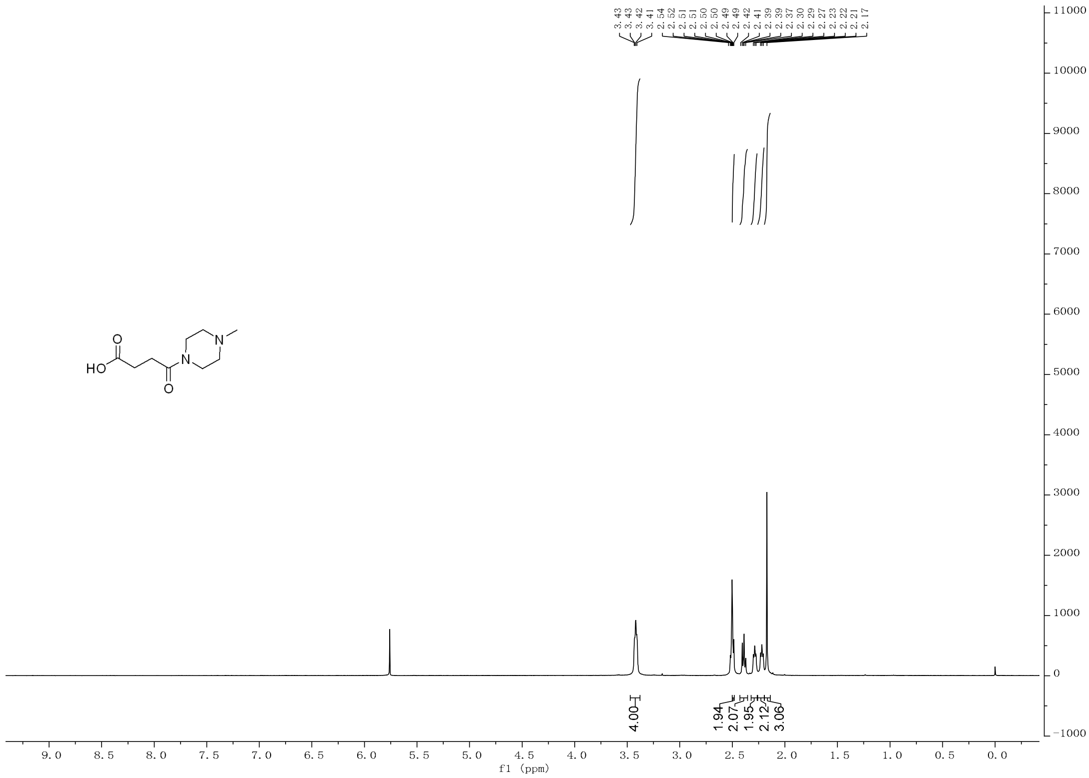

Figure S9. ^{1}H NMR spectrum for **2c** (DMSO-d_6, 400 MHz)

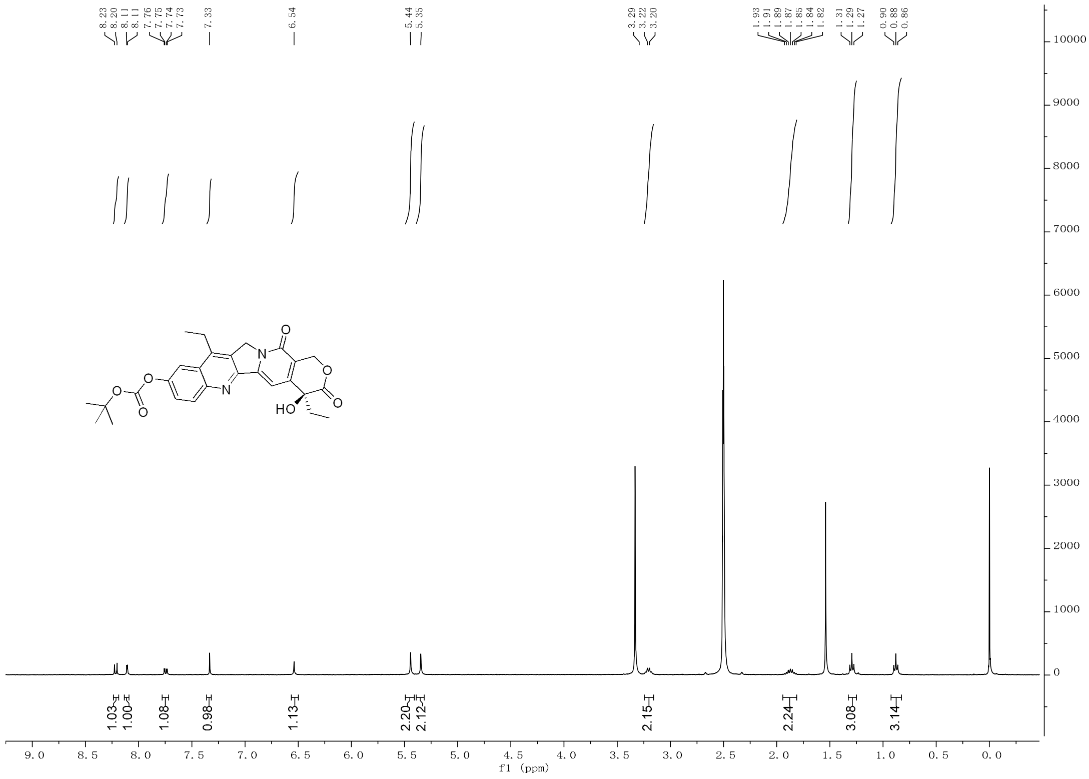

Figure S10. ^{1}H NMR spectrum for **3b** (DMSO-d_6, 400 MHz)

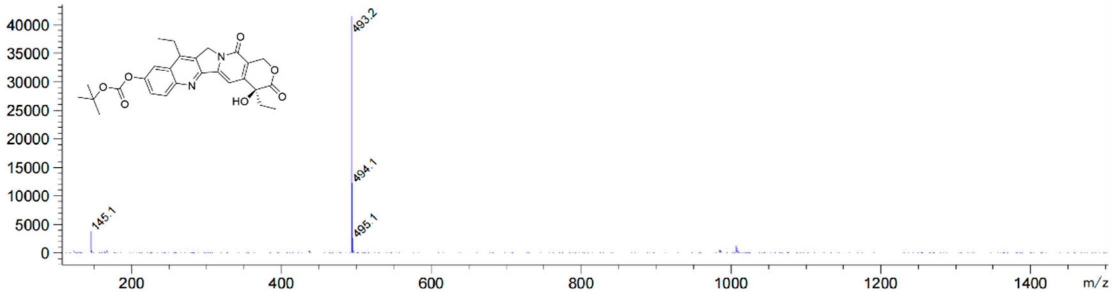

Figure S11. MS spectra of **3b**

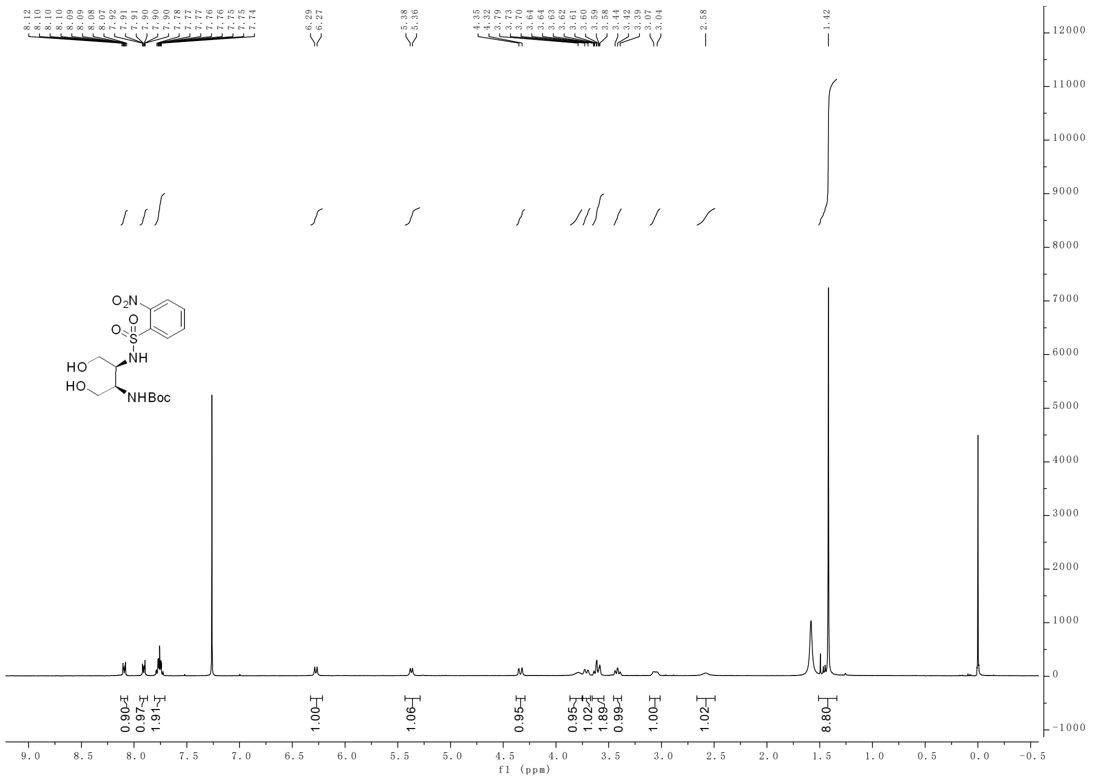

Figure S12. ^{1}H NMR spectrum for **4b** (DMSO-d_6, 400 MHz)

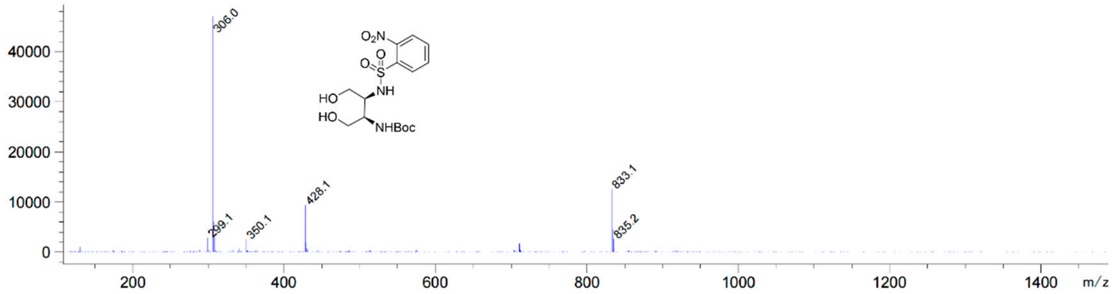

Figure S13. MS spectra of **4b**

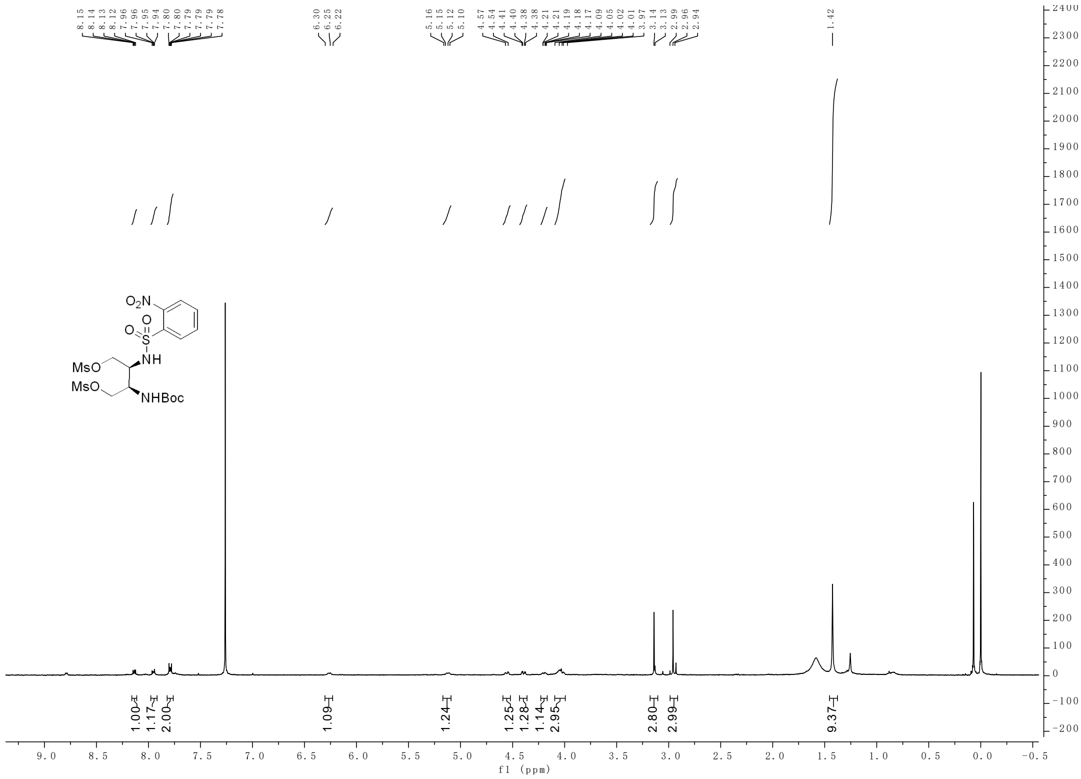

Figure S14. ^{1}H NMR spectrum for **4c** ($CDCl_3$, 400 MHz)

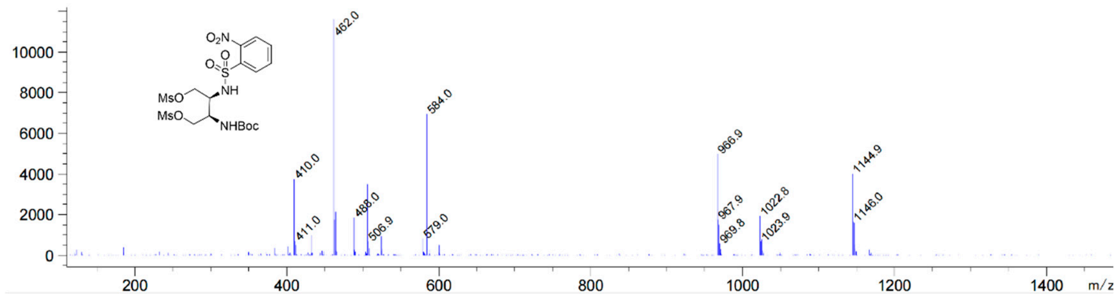

Figure S15. MS spectra of **4c**

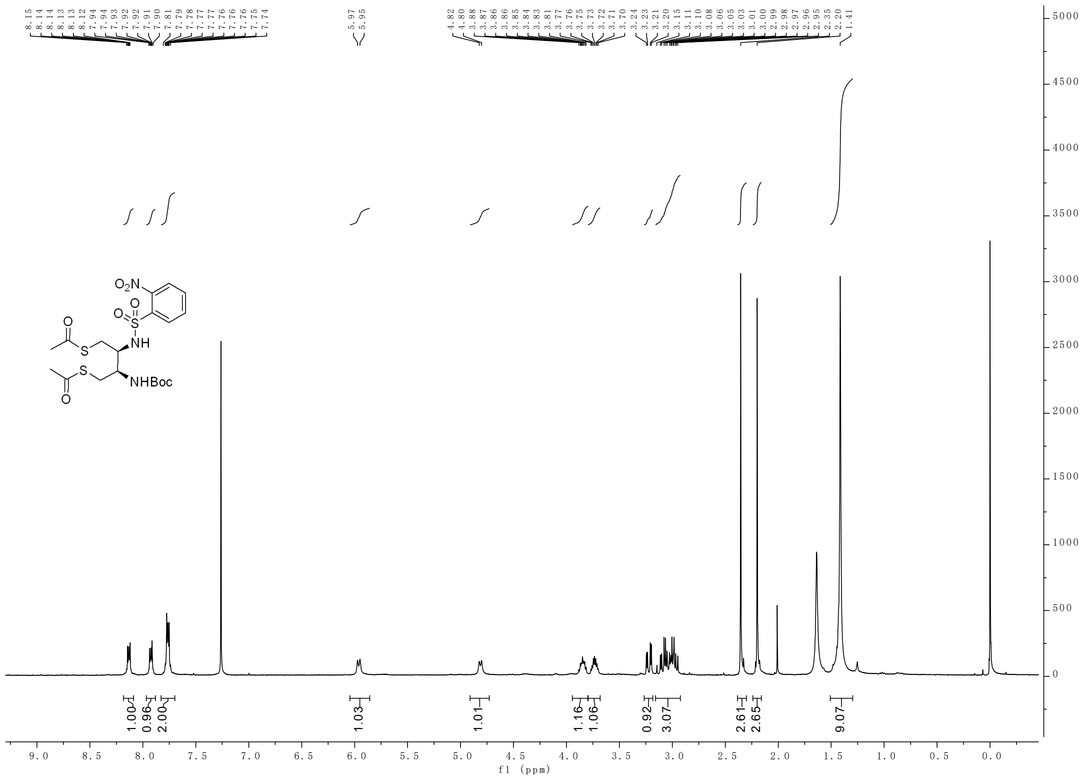

Figure S16. ^{1}H NMR spectrum for **4d** ($CDCl_3$, 400 MHz)

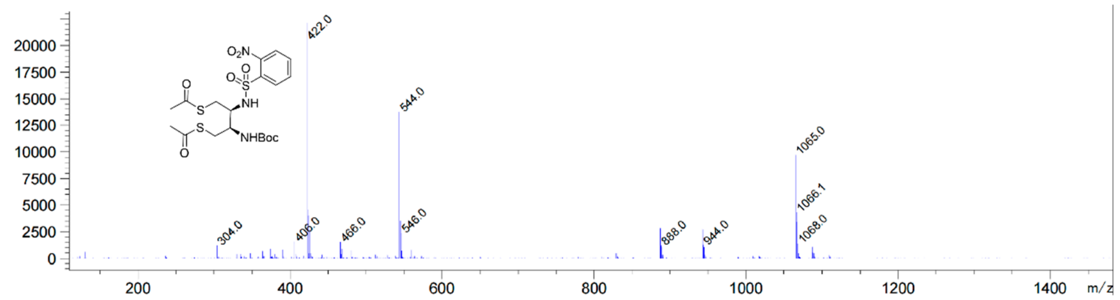

Figure S17. MS spectra of **4d**

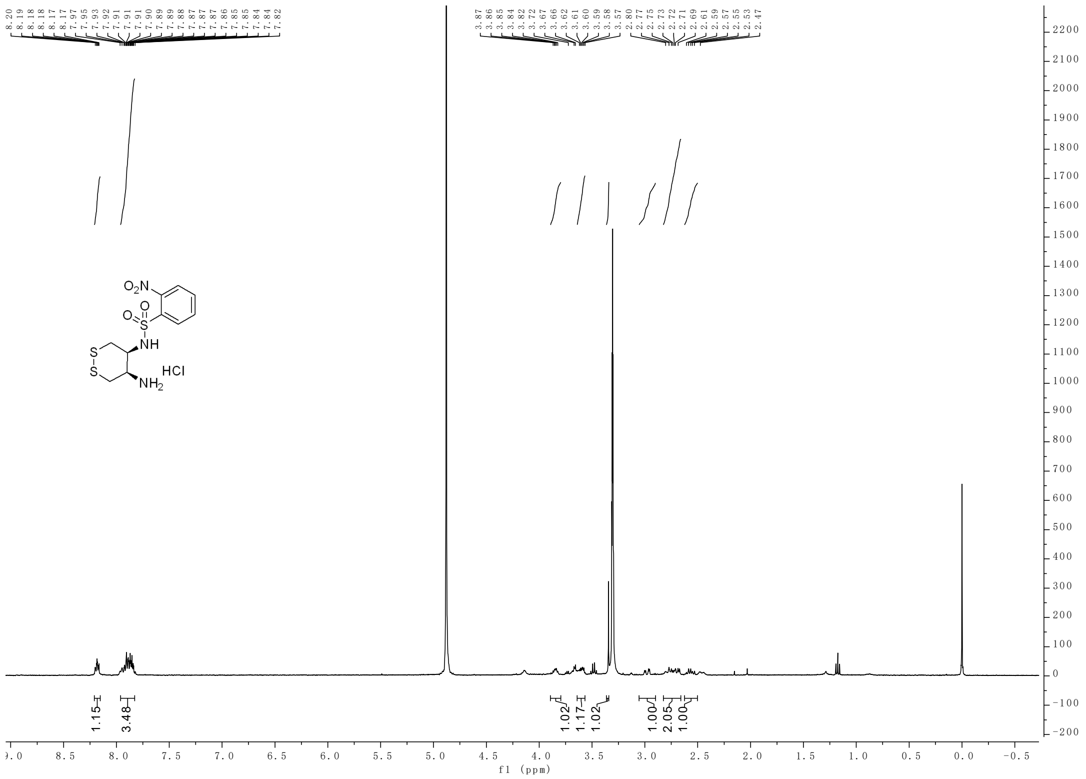

Figure S18. ^{1}H NMR spectrum for **4f** (Methanol-d_4, 400 MHz)

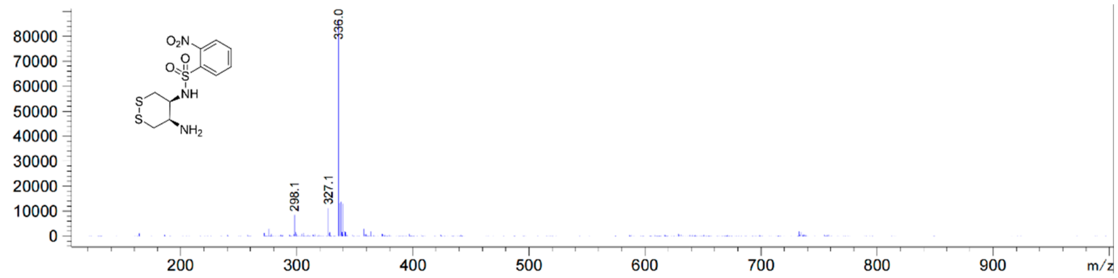

Figure S19. MS spectra of **4f**

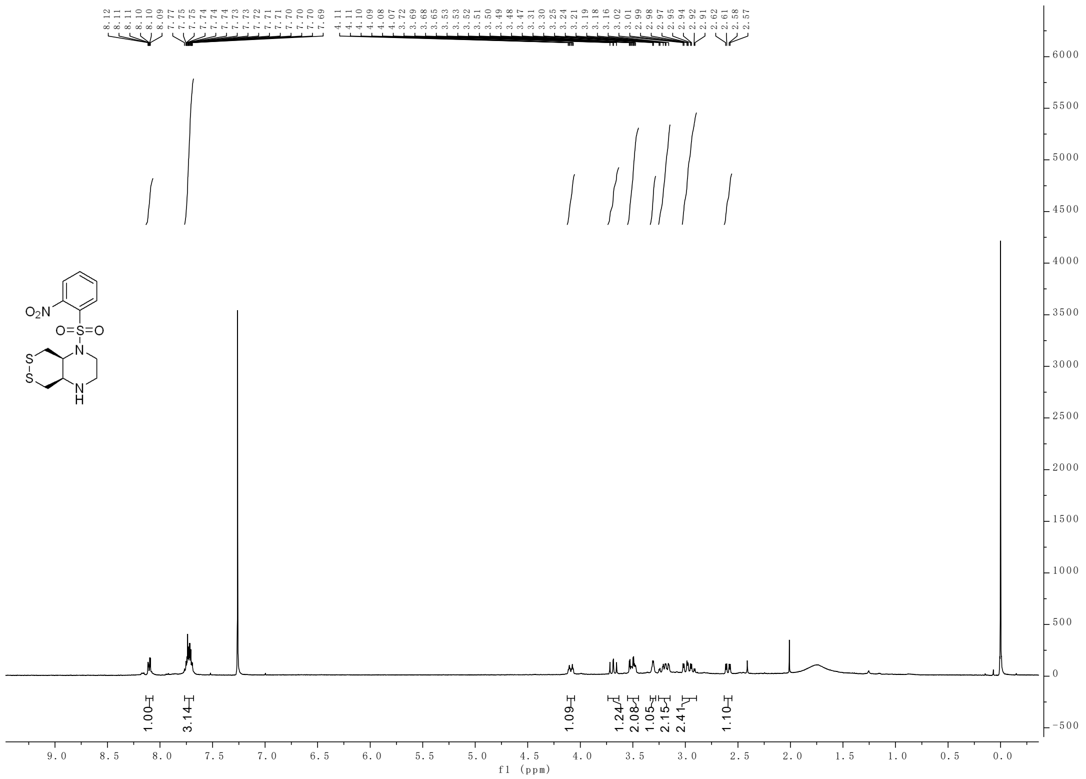

Figure S20. ^{1}H NMR spectrum for **4g** ($CDCl_3$, 400 MHz)

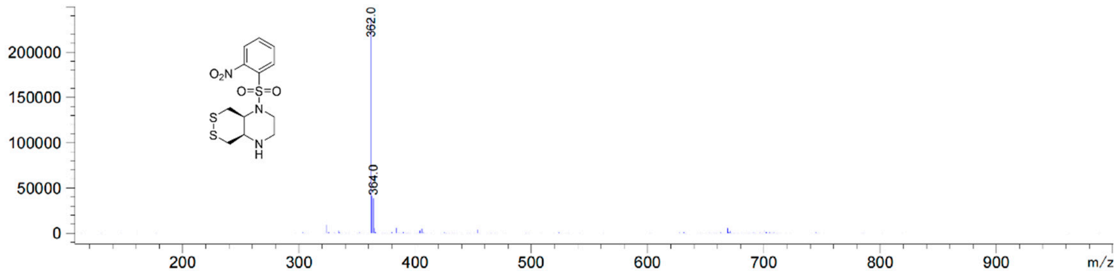

Figure S21. MS spectra of **4g**

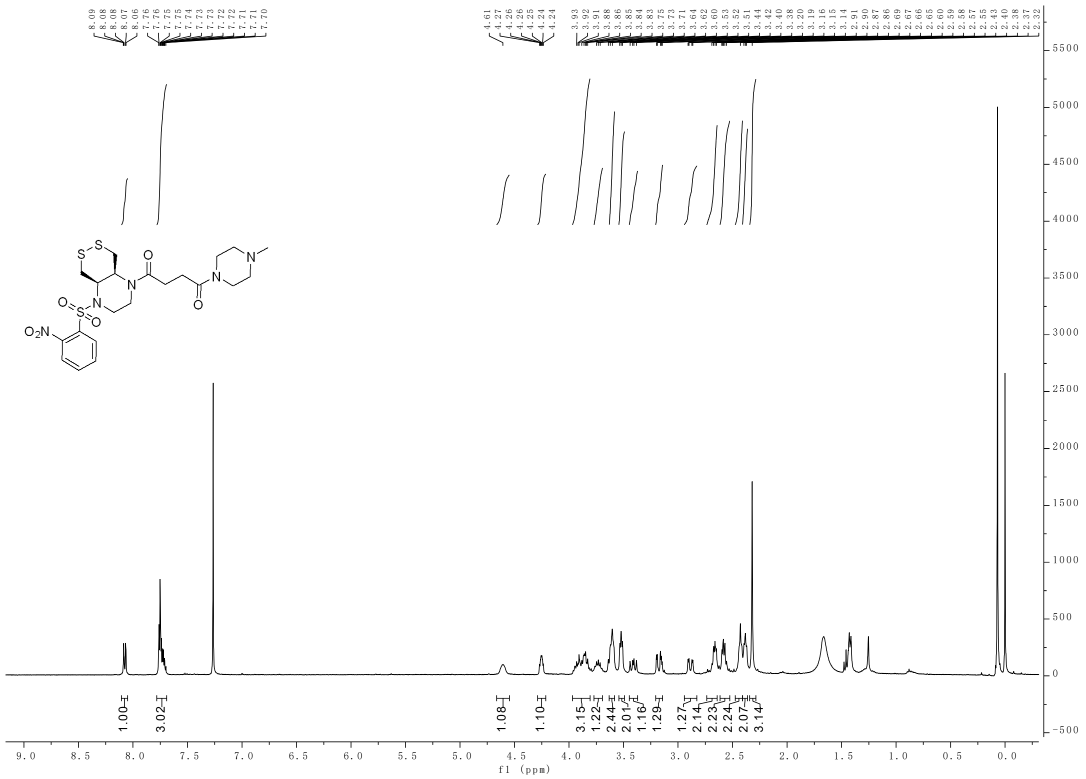

Figure S22. ^{1}H NMR spectrum for **4h** ($CDCl_3$, 400 MHz)

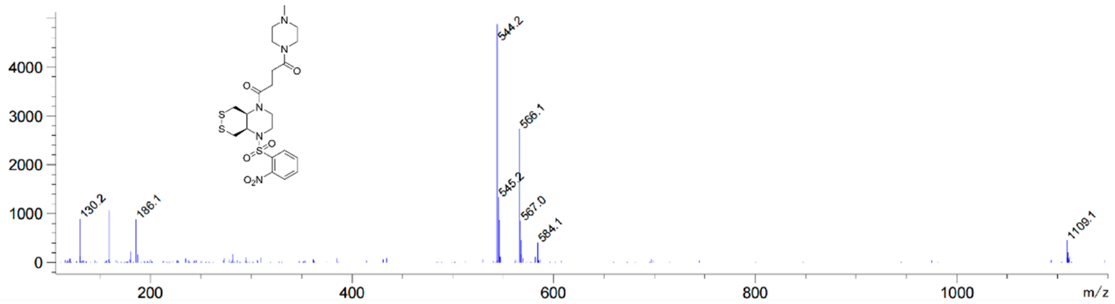

Figure S23. MS spectra of **4h**

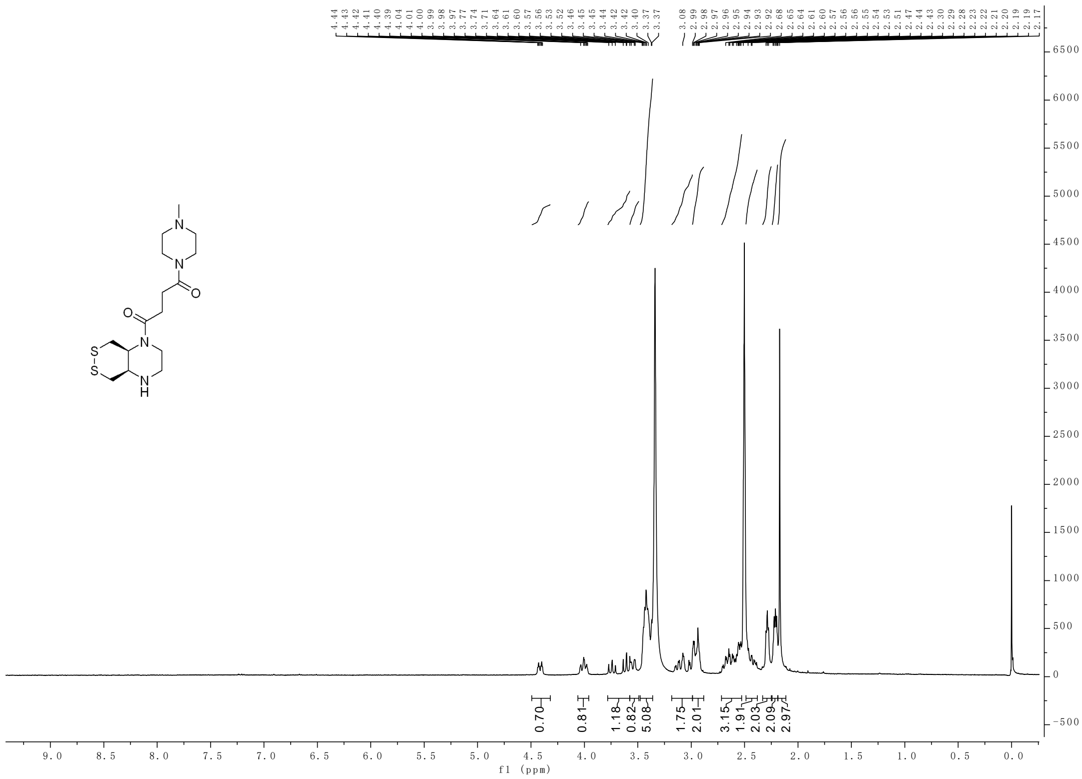

Figure S24. ^{1}H NMR spectrum for **4i** (DMSO-d_6, 400 MHz)

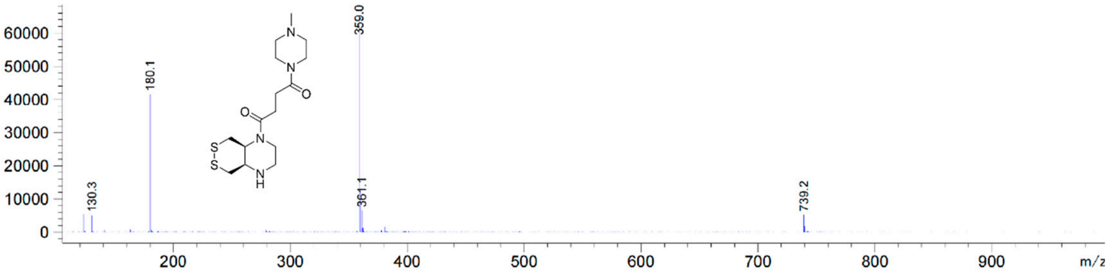

Figure S25. MS spectra of **4i**

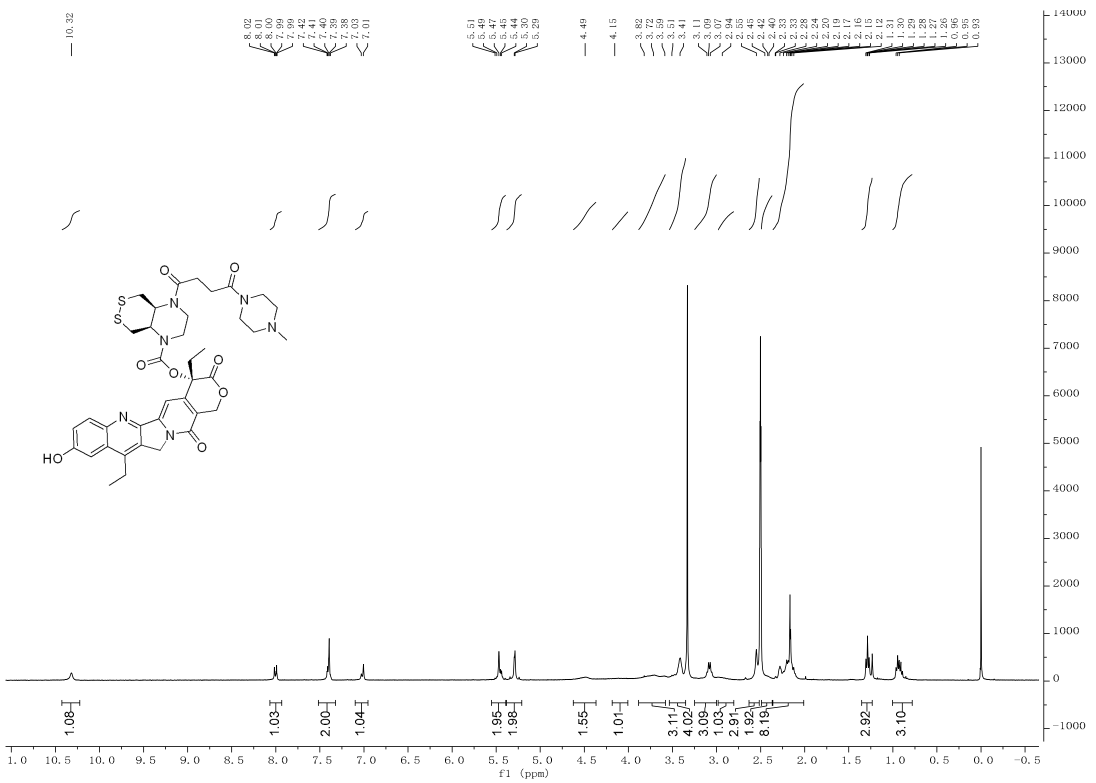

Figure S26. ^{1}H NMR spectrum for SN-38-CSS (DMSO-d_6, 400 MHz)

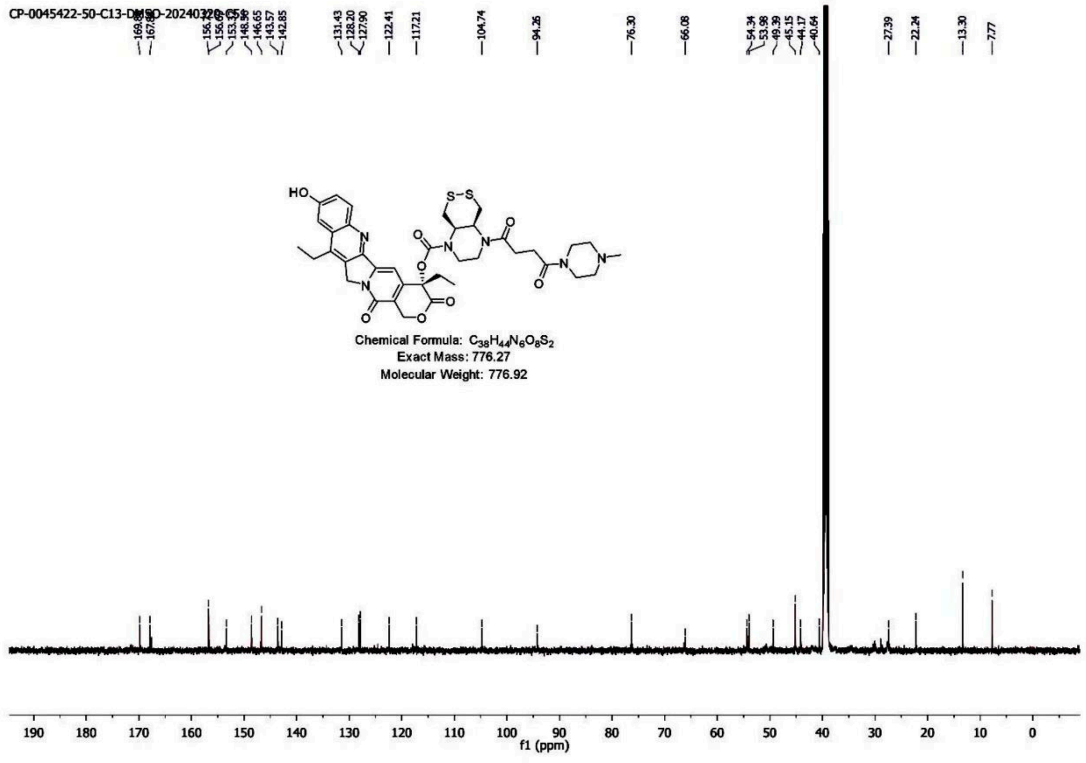

Figure S27. ^{13}C NMR spectrum for SN-38-CSS (DMSO-d_6, 100 MHz)

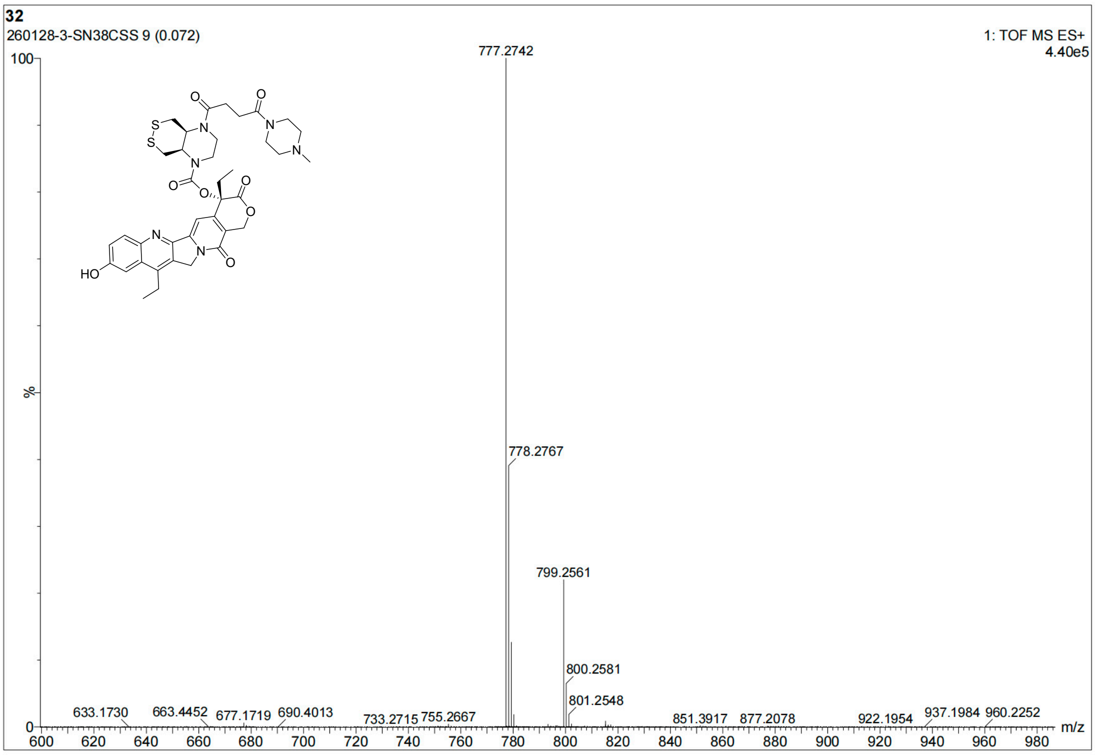

Figure S28. HRMS spectra of SN-38-CSS

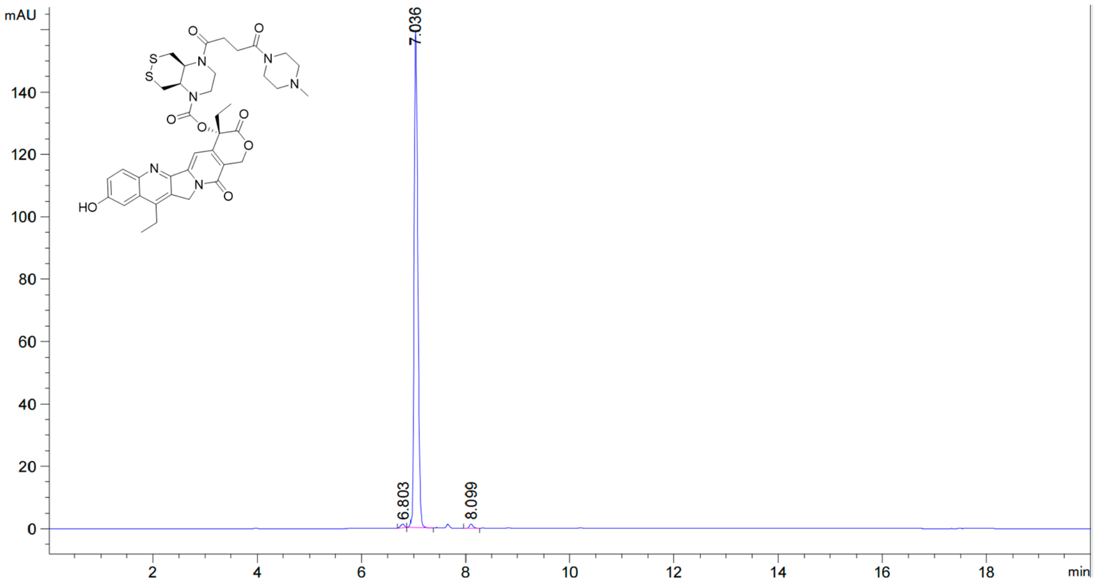

Peak #	Retention time [min]	Type	Peak width [min]	Peak area [mAU*s]	Peak height [mAU]	Peak area ratio %
1	6.803	BB	0.0781	6.12977	1.15102	0.7582
2	7.036	BB	0.0804	795.87158	159.01324	98.4431
3	8.099	BB	0.0704	6.45736	1.38483	0.7987

Figure S29. HPLC spectra of SN-38-CSS

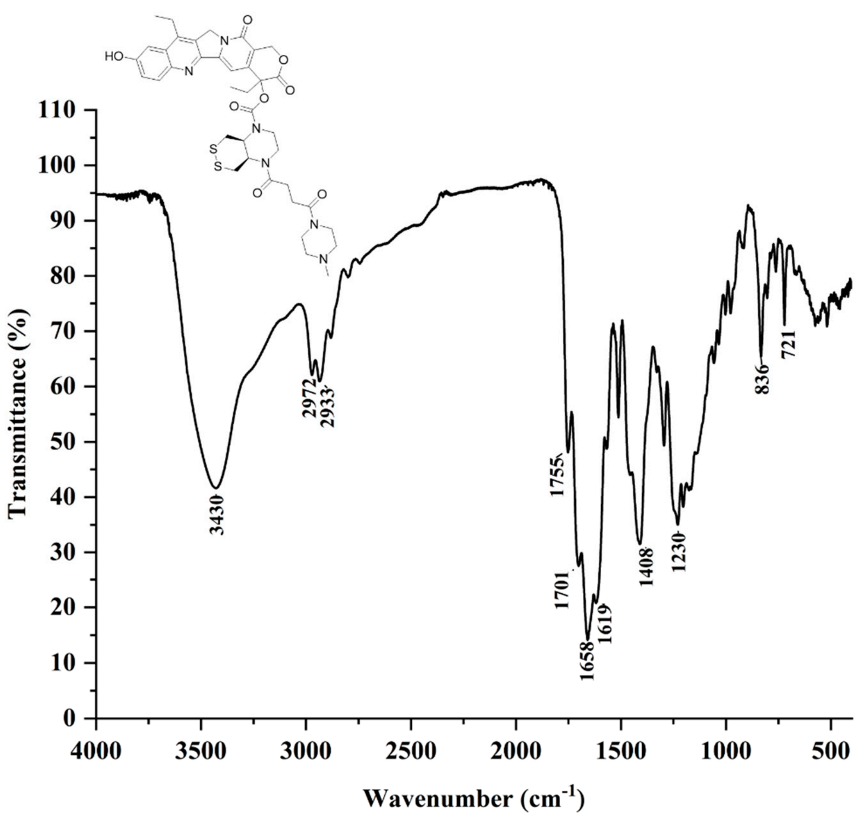

Figure S30. IR spectra of SN-38-CSS

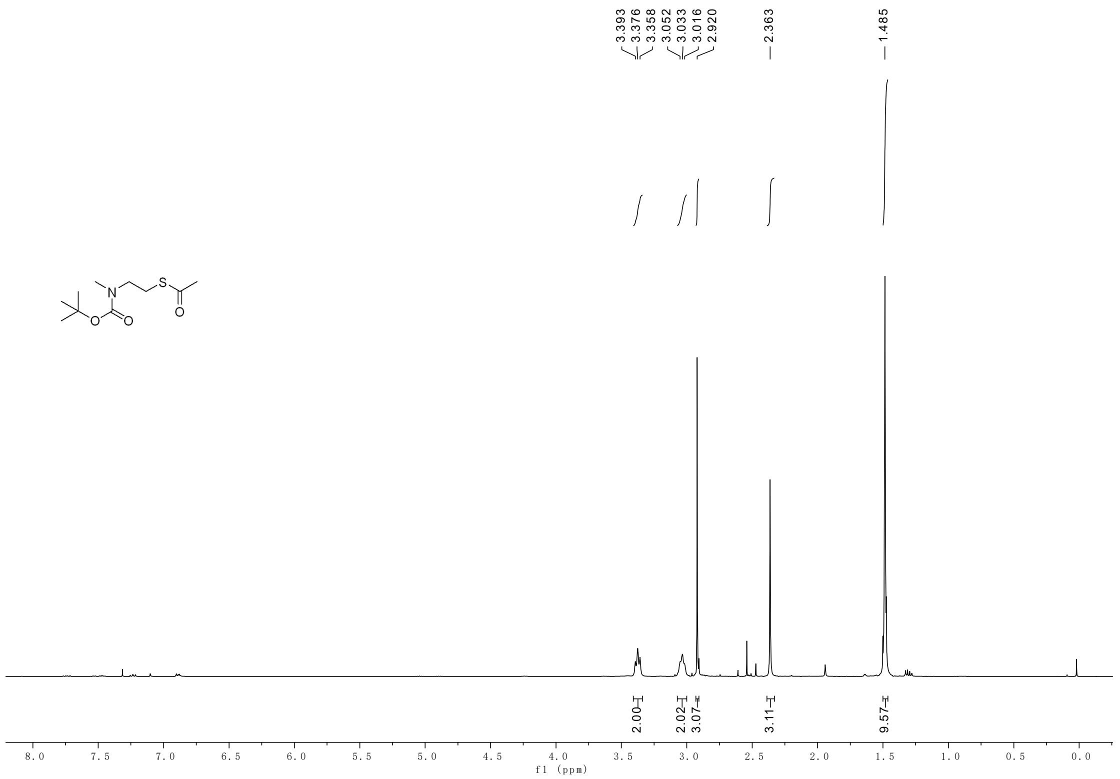

Figure S31. ^{1}H NMR spectrum for **5c** ($CDCl_3$, 400 MHz)

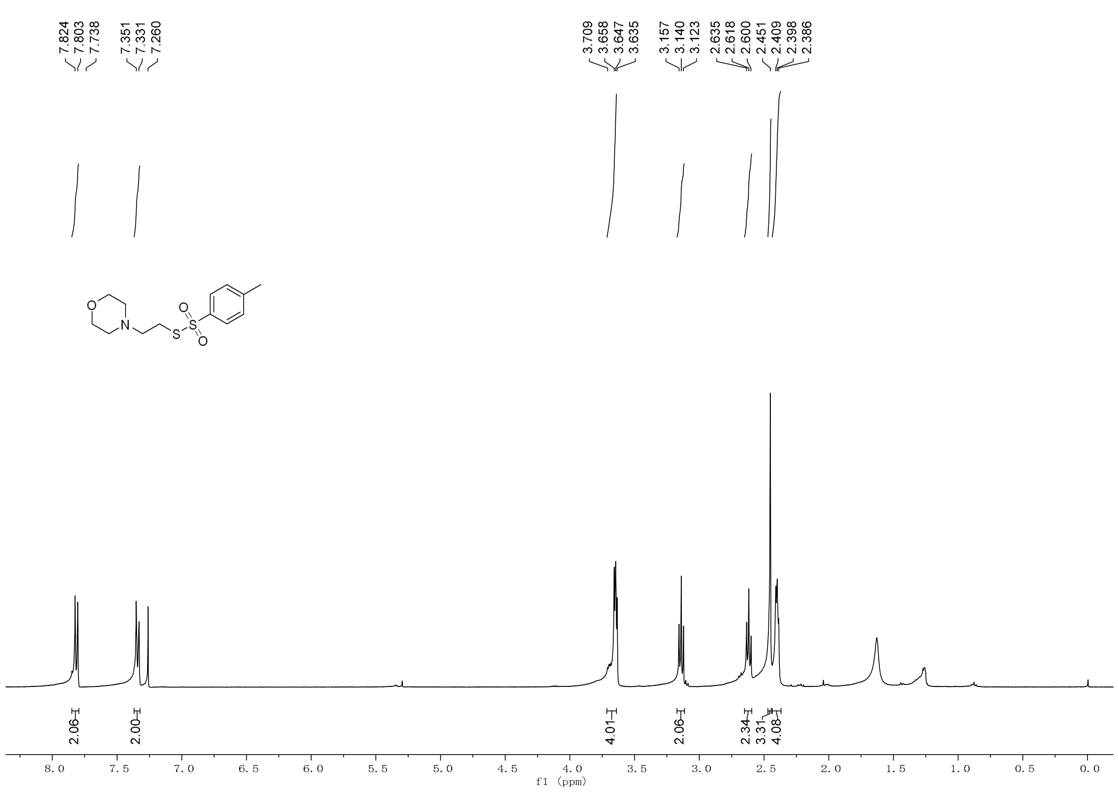

Figure S32. ^{1}H NMR spectrum for **6c** ($CDCl_3$, 400 MHz)

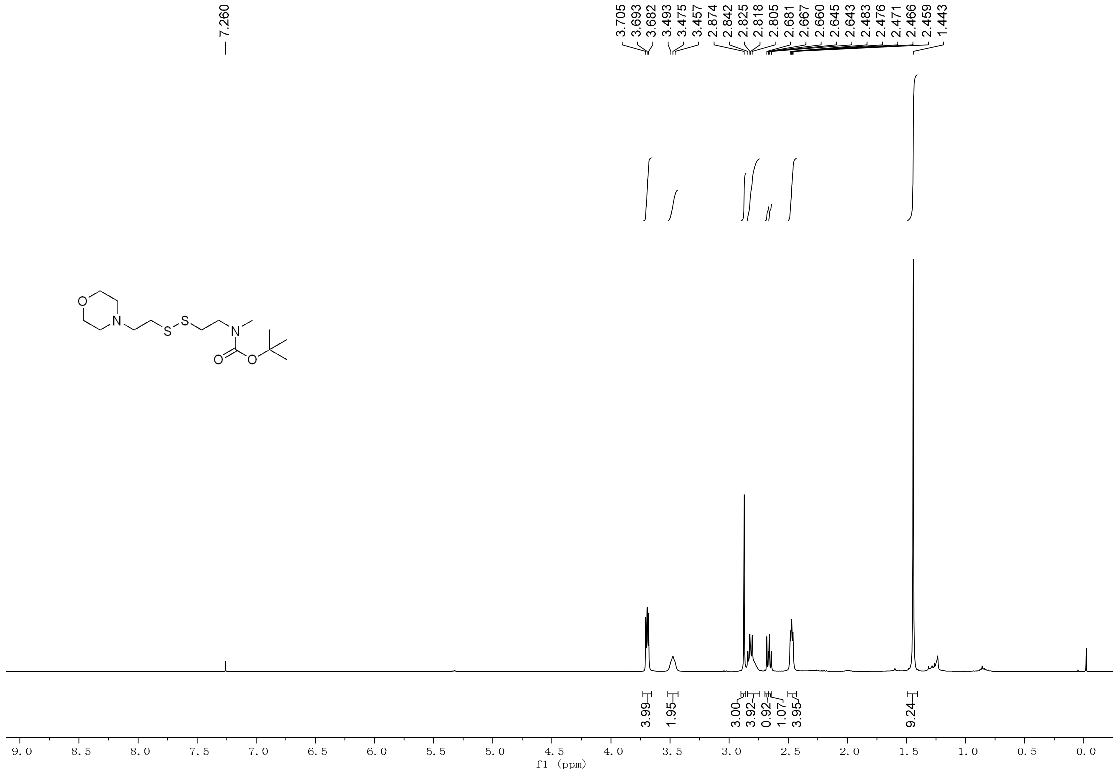

Figure S33. ^{1}H NMR spectrum for **6d** ($CDCl_3$, 400 MHz)

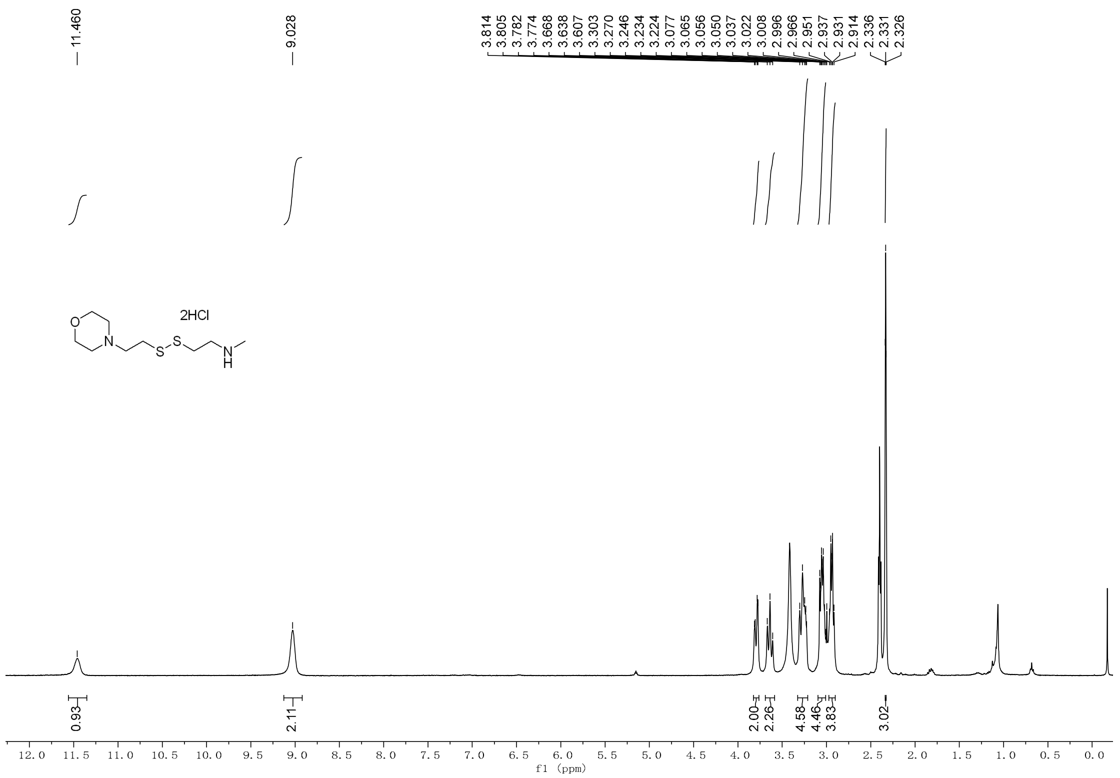

Figure S34. ^{1}H NMR spectrum for **6e** (DMSO-d_6, 400 MHz)

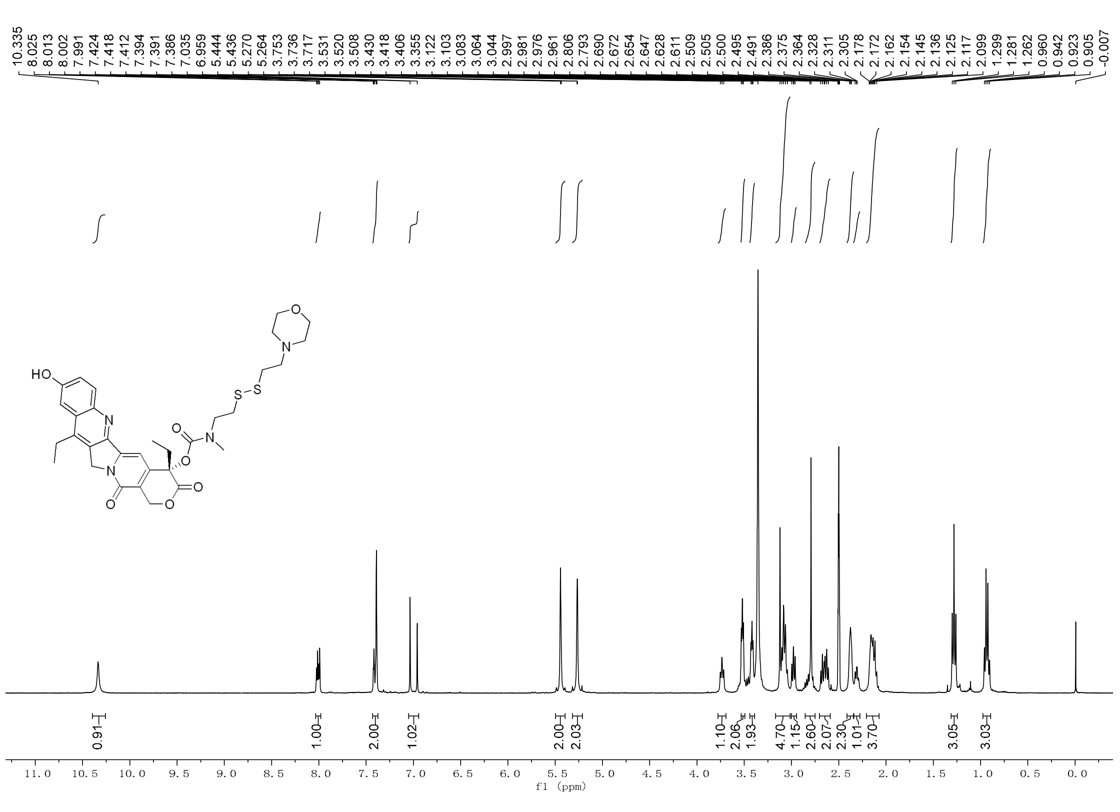

Figure S35. ^{1}H NMR spectrum for SN-38-LSS (DMSO-d_6, 400 MHz)

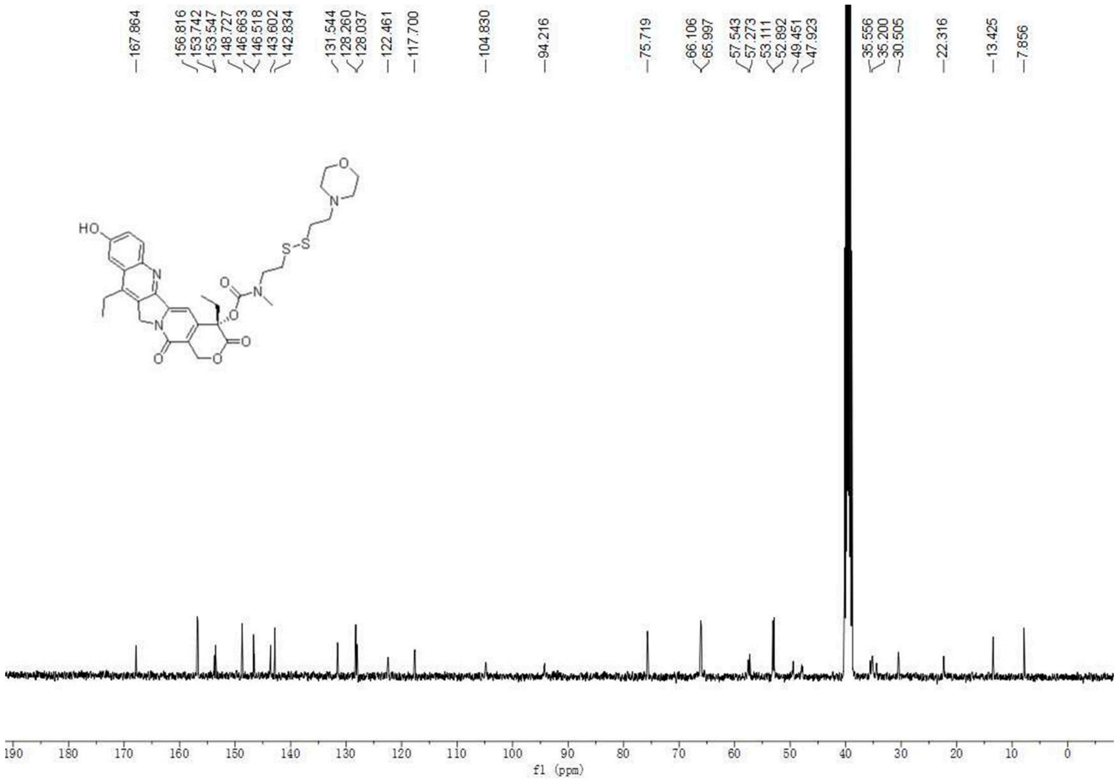

Figure S36. ^{13}C NMR spectrum for SN-38-LSS (DMSO-d_6, 100 MHz)

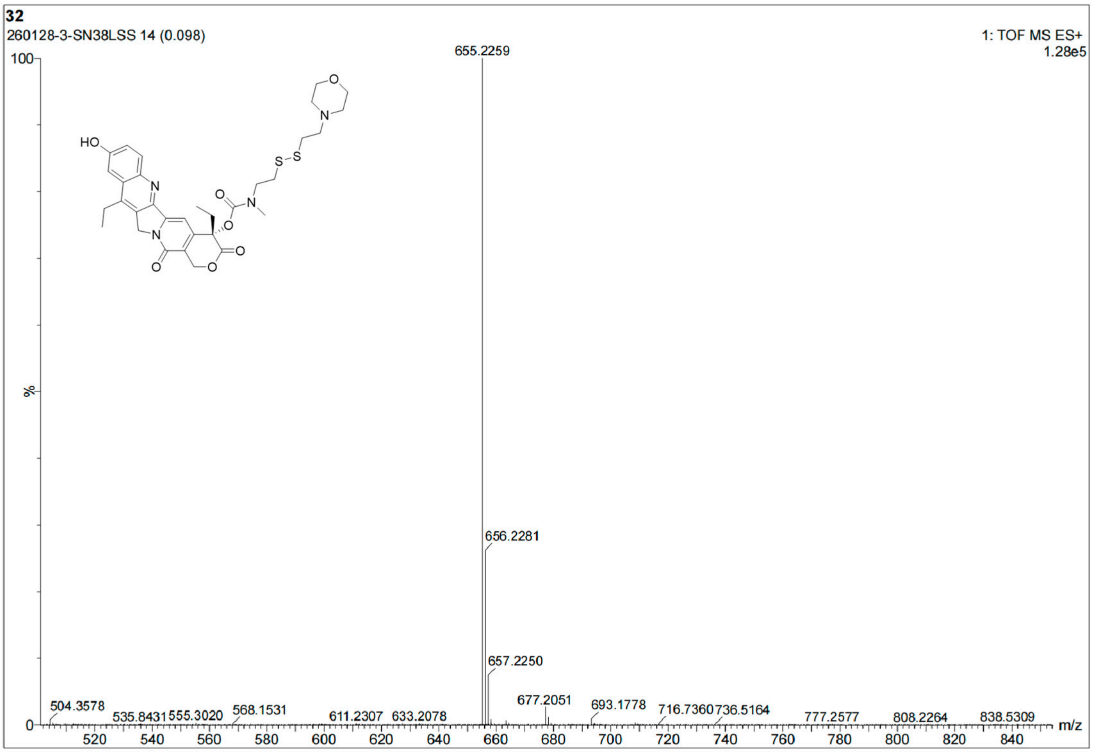

Figure S37. HRMS spectra of SN-38-LSS

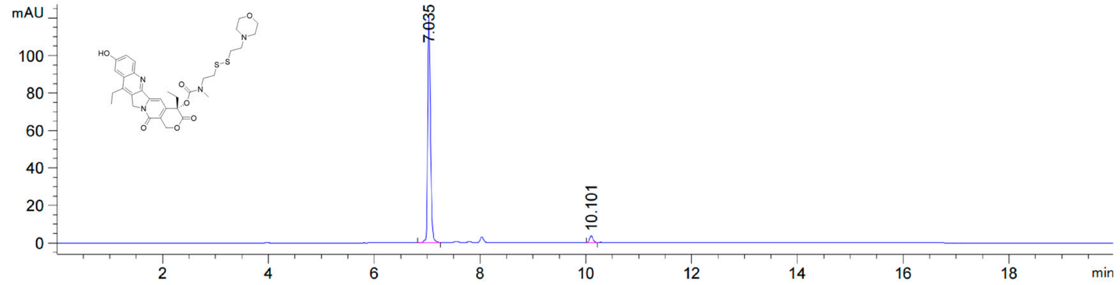

Peak #	Retention time [min]	Type	Peak width [min]	Peak area [mAU*s]	Peak height [mAU]	Peak area ratio %
1	7.035	BB	0.0570	447.55499	121.29946	96.4138
2	10.101	BB	0.0703	16.64708	3.71542	3.5862

Figure S38. HPLC spectrum for SN-38-LSS

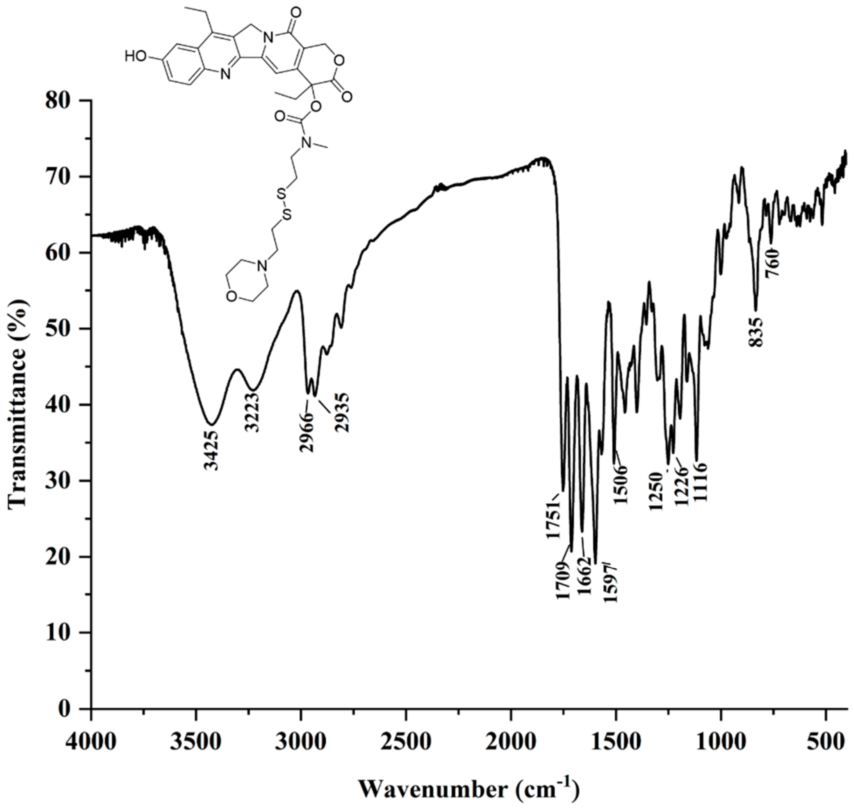

Figure S39. IR spectra of SN-38-LSS
